# Granulomatous cellular signatures in nontuberculous and tuberculous mycobacterial infections

**DOI:** 10.3389/fmicb.2025.1741883

**Published:** 2026-01-08

**Authors:** Brianna M. Doratt, Ethan G. Napier, Mahdi Eskandarian Boroujeni, Sarah Douglas, Michael H. Davies, Luiz Bermudez, Eliot R. Spindel, Erin F. McCaffrey, Ilhem Messaoudi

**Affiliations:** 1 Department of Microbiology, Immunology, and Molecular Genetics, College of Medicine, University of Kentucky, Lexington, KY, United States; 2 Spatial Immunology Unit, Laboratory of Parasitic Diseases, National Institute of Allergy and Infectious Disease, National Institutes of Health, Bethesda, MD, United States; 3 Division of Neuroscience, Oregon National Primate Research Center, Oregon Health and Science University, Beaverton, OR, United States; 4 Department of Microbiology, College of Sciences, Oregon State University, Corvallis, OR, United States

**Keywords:** aging lung, granuloma, macaque, mycobacterium, visium spatial transcriptomics

## Abstract

*Mycobacterium tuberculosis* (Mtb) and *nontuberculous mycobacteria* (NTM) are acid-fast bacilli that trigger granuloma formation, a hallmark immune response aimed at containing infection. While the biology of Mtb granulomas has been widely investigated, far less is known about granulomas caused by NTM infection, despite the increasing prevalence and clinical challenge of NTM cases worldwide. Because granulomas influence infection control, pathology, and treatment response, understanding their cellular organization and signaling is critical to improving therapeutic strategies. Therefore, we characterized granulomas formed following infection of rhesus macaques with *Mycobacterium avium* subsp. *hominissuis* (MAH) and compared them to granulomas from cynomolgus macaques infected with Mtb. MAH-associated granulomas were enriched in pro-inflammatory macrophages and, within granulomatous regions, both immune and non-immune cells exhibited pronounced inflammatory transcriptional signatures. By contrast, cells in non-granulomatous tissue displayed signatures associated with regulatory, wound-healing responses. Mtb granulomas were overall more inflammatory than MAH granulomas but showed diminished evidence of adaptive immune activation, cellular survival pathways, and signals supporting granuloma structural integrity. Notably, endothelial cells, fibroblasts, and a subset of macrophages were primary drivers of the signaling differences between Mtb and MAH granulomas. These findings highlight fundamental distinctions in granuloma biology between tuberculosis and nontuberculous infections. The reduced adaptive immune signatures and altered survival signaling seen in Mtb granulomas may underlie its aggressive pathology, while MAH granulomas’ distinct macrophage and stromal cell responses may contribute to the chronic, difficult-to-treat nature of this disease. Recognizing these differences could inform targeted therapies and strategies to better manage both tuberculous and nontuberculous infections.

## 1 Introduction

The clinical and immunological significance of granulomatous disease is underscored by its role as a hallmark and common manifestation of pulmonary infections caused by *Mycobacterium tuberculosis* (Mtb) and nontuberculous mycobacteria (NTM) (Ulrichs and Kaufmann, 2006), which collectively account for 1.25 million deaths annually worldwide (WHO, 2025). Granulomas constitute complex, highly organized aggregates of immune cells—predominantly macrophages—that sequester persistent pathogens within a fibrotic microenvironment, thereby limiting pathogen dissemination and enabling containment of organisms otherwise refractory to clearance by conventional immune effector mechanisms (Gil et al., 2010). The initial macrophage response to mycobacterial infection recruits additional monocytes, dendritic cells, neutrophils, T cells, and B cells, forming structured granulomatous lesions that serve both protective and pathological functions (Donovan et al., 2023). Oxygen depletion within granulomas leads to hypoxia and necrosis, profoundly affecting local immune cell function and tissue integrity (Hudock et al., 2017). Moreover, Mtb granulomas exhibit immunoregulatory properties that may limit effective bacterial clearance while enabling pathogen persistence and transmission (McCaffrey et al., 2022). The heterogeneity observed within granulomatous lesions across individuals and even within a single host highlights the complexity of host-pathogen interactions that govern these structures.

Although Mtb and NTM share similar mechanisms of infection, their virulence and epidemiology are markedly distinct. Unlike Mtb, which is highly transmissible via aerosolized droplets and remains a leading cause of global morbidity and mortality, NTM are not efficiently transmitted between humans and predominantly cause opportunistic infections (Warner and Mizrahi, 2007). As such, NTM disease occurs most frequently in immunocompromised individuals, older adults, and patients with chronic lung conditions such as emphysema or bronchiectasis (Gopalaswamy et al., 2020).

The first line of defense against mycobacterial infections is mediated by innate immune cells such as macrophages, dendritic cells, neutrophils, and natural killer cells (Sankar and Mishra, 2023). Phagocytic cells recognize mycobacteria via pattern recognition receptors, engulf the pathogen, and perform bactericidal activities (Byrne et al., 2015). These first responders also release cytokines such as IL-12, among others, that recruit additional immune cells and initiate the adaptive immune response (Cooper et al., 2011; Liu et al., 2005). CD4 + Th1 cells are crucial for mycobacterial control due to their ability to drive macrophage activation via IFNγ, and TNFα (Lo et al., 2021). The cytotoxic activity of CD8 + T cells is important for directly killing other infected cells (Lin and Flynn, 2015), while mucosal-associated invariant T cells provide rapid responses in the lung mucosa (Meermeier et al., 2022).

The engulfment of Mtb by alveolar macrophages (AM) triggers a type I immune response that recruits additional immune populations and initiates granuloma formation (Flynn et al., 2011). However, Mtb delivers virulence factors through the ESX-1 secretion system that disrupt host antimicrobial pathways, converting the granuloma into a specialized niche that supports bacterial persistence and immune evasion (Cambier et al., 2014). While Mtb-induced granulomatous disease has been extensively characterized (Flynn et al., 2011; Elkington et al., 2022; Sholeye et al., 2022; Qiu et al., 2024), the immunopathogenesis of NTM-associated granulomas remains poorly defined (Nakajima et al., 2021).

Animal models have been central to advancing our understanding of mycobacterial disease, yet each system presents limitations. Rodent models have been instrumental in studying Mtb, but do not recapitulate the structural complexity, cavitation, and fibrosis observed in humans (Corleis et al., 2023). Moreover, rodent inoculation with NTM typically results in disseminated disease rather than pulmonary disease (Rampacci et al., 2020). Clinical studies, while ideal, are constrained by limited access to infected lung tissues. In contrast, nonhuman primates (NHPs) have emerged as the gold-standard model for human Mtb infection and reliably reproduce granuloma heterogeneity and disease progression (Gideon et al., 2022). Importantly, recent work demonstrates that rhesus macaques also mirror key features of human NTM lung disease, providing a critical platform for dissecting host-pathogen interactions and informing therapeutic development (Napier et al., 2025; Winthrop et al., 2016; Cinco et al., 2024).

By leveraging Visium spatial transcriptomics, we comprehensively profiled *Mycobacterium avium* subsp. *hominissuis* (MAH)-associated granulomas and directly compared them to Mtb-mediated granulomas to delineate the immunological landscape underpinning granulomatous disease. MAH is a major species within the *M. avium* Complex (MAC), which accounts for the majority of pulmonary disease cases (Adjemian et al., 2012; Cowman et al., 2019; Henkle et al., 2015; Prevots et al., 2010; Winthrop et al., 2010). Our analysis unveiled a unique macrophage subset specifically enriched within granulomatous lesions. Additional comparison of tissue from MAH-infected (right) and uninfected (left) lungs demonstrated that both immune and non-immune cells adjacent to granulomas participate actively in inflammation and antimicrobial responses, underscoring the complex multicellular orchestration within the granuloma microenvironment. Notably, lung tissue from the uninfected lung displayed a pronounced M2-like regulatory signature. Strikingly, comparison of MAH- and Mtb-induced granulomas revealed that both immune and parenchymal cells were more proinflammatory in Mtb lesions, with transcriptomic data predicting a profound disruption of pathways critical for adaptive immunity activation and granuloma maintenance. Mtb granulomas also exhibited enhanced fibronectin signaling but diminished macrophage survival and impaired regulation of cell migration and adhesion, providing new insight into the mechanisms of disease persistence and immune evasion in tuberculosis.

## 2 Materials and methods

### 2.1 Ethics statement

Animal work was performed in accordance with the recommendations described in the Guide for the Care and Use of Laboratory Animals of the National Institute of Health, the Office of Animal Welfare and the Animal Welfare Act, United States Department of Agriculture. The rhesus macaque studies with MAH were approved by the Institutional Animal Care and Use Committees (IACUC) at the Oregon National Primate Research Center.

### 2.2 Cohort description, animal infection, and sample collection

Two young (5–7 years; 1 female, 1 male) colony-bred Indian-origin rhesus macaques (*Macaca mulatta*) were intrabronchially inoculated in the right upper, middle, and caudal lobe with a total of 6.8×10^8^ CFU of *M. avium* subsp. *hominissuis* strain 101 (MAH) divided equally between the 3 inoculation sites (Napier et al., 2025). MAH was obtained from a patient with AIDS and shown to be virulent in mouse models (Bermudez et al., 2008). The bacteria were grown on Middlebrook 7H10 agar. The viability of the inoculum was 94 +/− 2% determined as previously described (Bermudez et al., 2008). The left and right cranial tissue of two young animals (Y1 and Y2), as well as the right caudal tissue of Y2 was collected at necropsy (310 days post infection) and was subjected to first generation Visium spatial transcriptomics. Formalin-fixed paraffin-embedded (FFPE) pulmonary tissue from a cynomolgus macaque infected with Mtb (9.3 CFU) via bronchoscopic inoculation and followed for 61 days before necropsy was analyzed alongside the MAH infected tissue (McCaffrey et al., 2025).

### 2.3 Immunohistochemistry and immunofluorescence analysis

Immunohistochemistry staining of the MAH infected tissue was done on the Ventana Discovery Ultra platform. 4 μm FFPE sections were cut & mounted onto plus charged slides that were dried overnight at 60 °C. Deparaffinization was done before antigen retrieval with Ventana CC1 buffer for 48 min. Myeloperoxidase (predilute, Roche 05267692001) was added at 37 °C for 32 min. Post-primary peroxidase quench was done with Discovery Inhibitor (Ventana 760–4,840) for 4 min at 37 °C. Slides were incubated with Discovery Ultramap anti-Rabbit-AP (Ventana 760–4,314) for 12 min at 37 °C followed by discovery Red Chromogen Kit (Ventana 760–228) for 4 min at 37 °C, CD68 KP1 (predilute, Dako IR609) for 60 min at 37 °C, anti-Mouse HQ (Ventana 760–4,814) 20 min at 37 °C, anti-HQ-HRP for 20 min at 37 °C, Discovery Teal HRP Kit (Ventana 760–247) for 12 min. Antibody denaturation was done with Ventana CC2 (Ventana 950–223) at 100 °C. Slides were then incubated with Dako Dual (AP/HRP) Endogenous Block (Agilent K4065) for 16 min at 37 °C followed by CD3 2GV6 (Ventana 790–4,341), Omni-map anti-rabbit-HRP (Ventana 760–4,311), DAB (Ventana 760–159), CD20 (Ventana 760–2,531), Ultramap anti-mouse-AP (Ventana 760–4,312), and Discovery Yellow (Ventana 760–239) all at 37 °C for 20 min. Counterstain was done with Mayer’s hematoxylin for 5 min. Slides were air dried overnight before permanent mounting. All runs included a human tonsil as control for staining.

Immunofluorescent staining of the MAH infected tissue was done on 5-micron thick sections of FFPE macaque lung sections placed onto glass slides and baked at 60 °C for 1 h and deparaffinized. Heat induced antigen retrieval using the RNAScope Target Retrieval buffer (ACDBio Cat#322000) was then performed in a Biocare Decloaking Chamber at 110 °C for 15 min. Once the decloaking chamber was cooled to 95 °C the slides were taken out of the chamber and left to cool at room temperature for 20 min. Slides were then washed in dH2O and TBS-T before overnight incubation with a mouse anti-CD3 antibody (LN10 Biocare ACI B3152C) at room temperature. Slides were then washed in 1X TBS-T, treated with 3% H_2_O_2_ in PBS for 10 min, and incubated with the Gold Bridge International Labs Polink 1 HRP Detection System against Mouse IgG (D12-110) for 20 min at room temperature. CD3 staining was visualized with the CF488 tyramide fluorophore (Biotium 92,171). The attached mouse anti-CD3 antibody and Polink 1 secondary antibody were stripped off the tissue section by boiling in ACDBio Target Retrieval buffer for 15 min. The slides were then incubated with a mixture of the Goat anti-CD20 (Invitrogen PA1-9024), mouse CD68/163 (CD68 Biocare CM033C; CD163 Thermo MA5-11458) and rabbit anti-MPO (Agilent A039829-2) antibodies overnight at room temperature. The next day the slides were washed and incubated with secondary antibodies against mouse IgG (AF568 – Thermo Fisher A10037), Goat IgG (AF647 – Thermo Fisher A21447) and rabbit IgG (Dy755 – Thermo Fisher SA5-10043) for 2 h at room temperature. The slides were then counterstained with DAPI and coverslipped with the Prolong Gold mounting media (Thermo Fisher P36930). Stained slides were imaged using a Zeiss Axioscanner at 20X. To demonstrate stripping efficiency, staining of control lung and lymph node slides with only anti-CD3 antibody plus all secondary antibodies were concurrently performed and imaged at the same exposure rate as the rest of the samples.

### 2.4 Visium spatial transcriptomics library preparation

The impact of MAH infection on gene expression in the context of lung architecture as well as the differences between the MAH and Mtb granulomas were assessed by first generation Visium spatial transcriptomics (10x Genomics) with CytAssist using Demonstrated Protocol CG000520. Briefly, for MAH granulomas, a 20 μm scroll from an FFPE block was deparaffinized and fixed RNA was extracted using the Qiagen FFPE RNA kit per manufacturers protocol. Extracted RNA was run on the Agilent Bioanalyzer using the pico RNA chip for DV200 determination. RNA integrity and DV200 values were first collected to ensure high quality of initial samples (Supplementary Table S1). Serial sections from blocks with a DV200 > 40% were mounted on a microscope slide and stained with hematoxylin and eosin. Following imaging, slides were de-crosslinked and immediately hybridized with the Visium Human Transcriptome Probe Kit v2 (PN-1000466, 10x Genomics) as per manufacturer instructions (10x Genomics, User Guide CG000495). Approximately 80% of the human probe set align with the appropriate macaque homolog. Ligated probes were captured on Visium slides using a CytAssist instrument (10x Genomics) and probe libraries were amplified as per kit instructions. Libraries were sequenced by Novogene using the Illumina NovaSeqX with a sequencing target of 25,000 paired reads per covered spot.

### 2.5 Visium spatial transcriptomics analysis

Data obtained from this study was combined with Visium spatial transcriptomic data obtained from an Mtb granuloma from a cynomolgus macaque (McCaffrey et al., 2025). For Visium spatial transcriptomic library data preprocessing, raw reads were aligned and quantified using the SpaceRanger Software Suite (Version 3.1.1, 10X Genomics) against the GRCh38 human reference genome and probe set V2. Downstream processing of aligned reads was performed using Seurat (Version 5.1.0) in R (Version 4.1.1). Data normalization and variance stabilization were performed on the integrated object using the NormalizeData and ScaleData functions in Seurat, before merging the data using Harmony. Dimensional reduction was performed using the RunPCA function to obtain the first 30 principal components and clusters visualized using Seurat’s RunUMAP function with a resolution of 0.5. Cell types were assigned to individual clusters using FindAllMarkers function with a log2 fold change cutoff of at least 0.4, FDR < 0.05, and using the EnrichR cell type database (Xie et al., 2021) (Supplementary Tables S2, S3). Differential gene expression analysis was performed using QLF pseudobulk function in EdgeR and differentially expressed genes were defined as having an FDR < 0.05 and a log2 fold change ± 0.4. Functional enrichment was performed using Metascape (Zhou et al., 2019) (Supplementary Tables S4–S6). The CellChat package was used to investigate possible differential ligand-receptor pair interactions between tissue from the left and right lung sections (Jin et al., 2021) (Supplementary Tables S7, S8). Differential trajectory analysis was performed using slingshot with the Condiments package (v1.17). Temporally expressed genes were identified by ranking all genes by their variance across pseudotime and then further fit using the generalized additive model with pseudotime and infection as independent variables.

## 3 Results

### 3.1 Spatial transcriptomic profiling of MAH granulomatous tissue revealed a distinct disease-specific macrophage population

**Figure 1:**
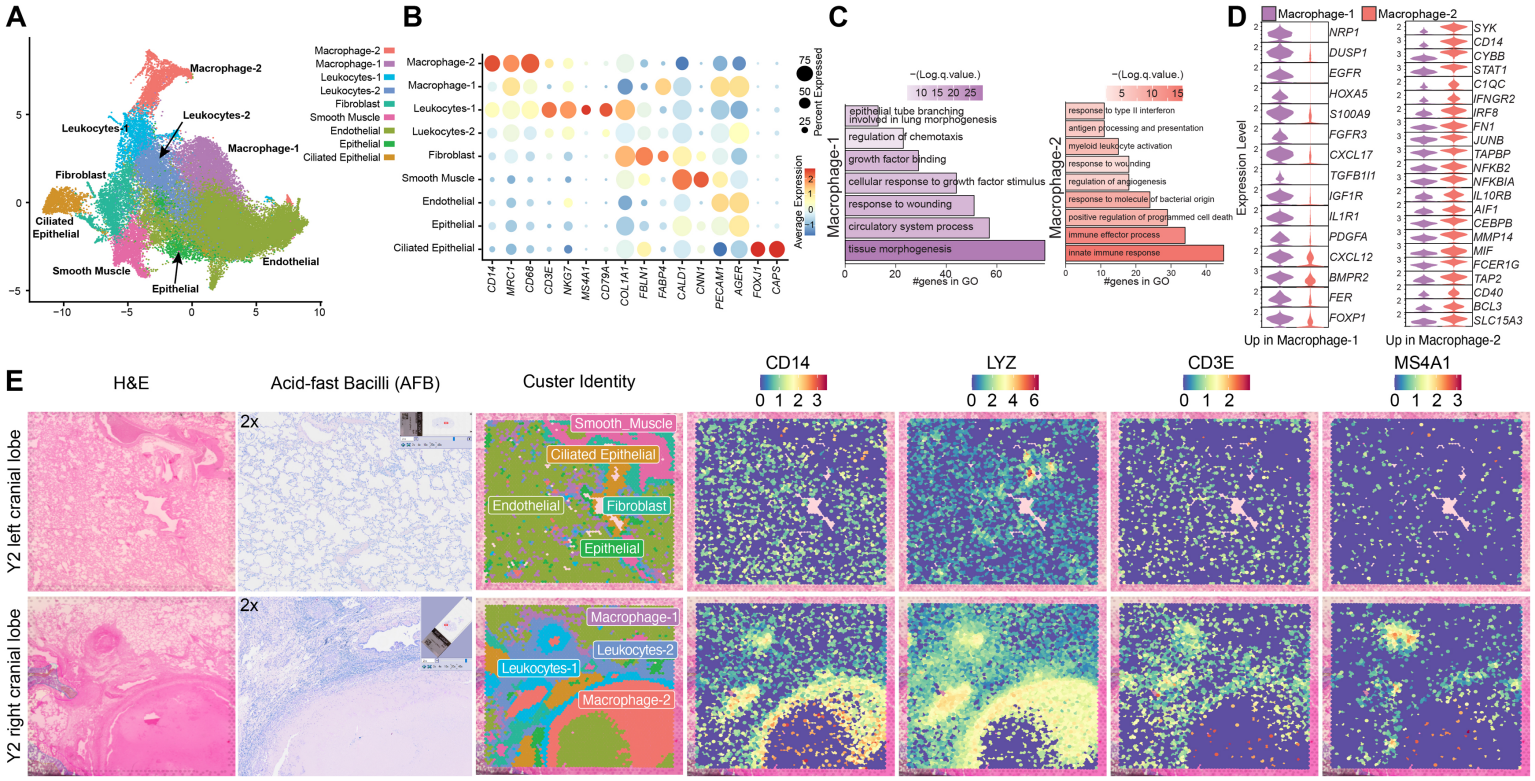
Granulomatous lung regions are distinct from other lung regions. **(A)** UMAP of 22,286 spots from left and right lung tissue sections of young MAH infected animals. **(B)** Bubble plot of key marker genes used to identify cell populations in panel A. The size of the bubble denotes the percent of cells expressing the marker and color denotes the average expression level of the marker. **(C)** Bar plot of GO terms for DEGs upregulated in the macrophage-2 group relative to the macrophage-1 group in the right lung of young animals. Length of the bar indicates the number of genes mapping to each GO term, and the intensity of color denotes the -log10(*Q*) value. **(D)** Violin plots depicting the differentially expressed genes (DEG) between the macrophage-1 and macrophage-2 clusters in the right lungs of young animals. **(E)** Representative image of H&E stained left and right lung tissue sections, acid-fast bacilli stain, and H&E overlayed with cluster identity spots and expression of indicated genes (*CD14, LYZ, CD3E, MS4A1)*.

H&E and immunostaining showed presence of granulomatous regions with accumulation of macrophages, T cells, B cells, and neutrophils in the right but not left lungs which, had minimal immune cell infiltration within the thin and open alveolar spaces (Supplementary Figure S1). We delineated 9 transcriptionally defined clusters based on expression of canonical marker genes and functional enrichment analyses of cluster-defining genes using Enrichr (Xie et al., 2021) (Supplementary Figure S2A). Both leukocytes-1 (*CD3E, NKG7, MS4A1, CD79A*) and leukocytes-2 (*CD3E, CD79A*) were predominantly composed of T and B cells (Figures 1A,B; Supplementary Figure S1B). Additionally, we note the presence of macrophage-1 (*CD14, MRC1, CD68*) as well as a unique granuloma-specific macrophage subset, characterized by increased expression of *CD14, MRC1,* and *CD68* expression (macrophage-2 cluster) (Figures 1A,B; Supplementary Figure S1B). Other subsets include fibroblasts (*FBLN1, FABP4*), smooth muscle (*CALD1, CNN1*), endothelial (*PECAM1, AGER*), epithelial (*PECAM1*low, *AGER*), and ciliated epithelium (*FOXJ1, CAPS*) (Figures 1A,B; Supplementary Figure S1B).

Comparative profiling of macrophage subclusters revealed that the macrophage-1 cluster expressed higher levels of genes important for tissue repair and epithelial support (*EGFR, FGFR3*) (Figures 1C,D), whereas macrophage-2 upregulated host-defense mediators (*SYK, CD14, CYBB*) (Figures 1C,D). Lung sections containing granulomas had higher frequency of leukocytes-1, leukocytes-2, and macrophage-2 subsets that was accompanied by a reduction in macrophage-1, smooth muscle and endothelial populations compared non-infected lung sections (Supplementary Figure S2C). Spatial mapping revealed a macrophage-dense core encircled by leukocytes-1 and -2, consistent with canonical granulomatous architecture, but with no evidence of mycobacteria via acid-fast staining (Figure 1E; Supplementary Figure S1D). Leukocytes-1 were localized immediately around macrophage-2 whereas leukocytes-2 were localized on the outskirts of the granuloma (Figure 1C; Supplementary Figure S1D).

### 3.2 Divergent immune activation and tissue repair programs define granulomatous inflammation

**Figure 2:**
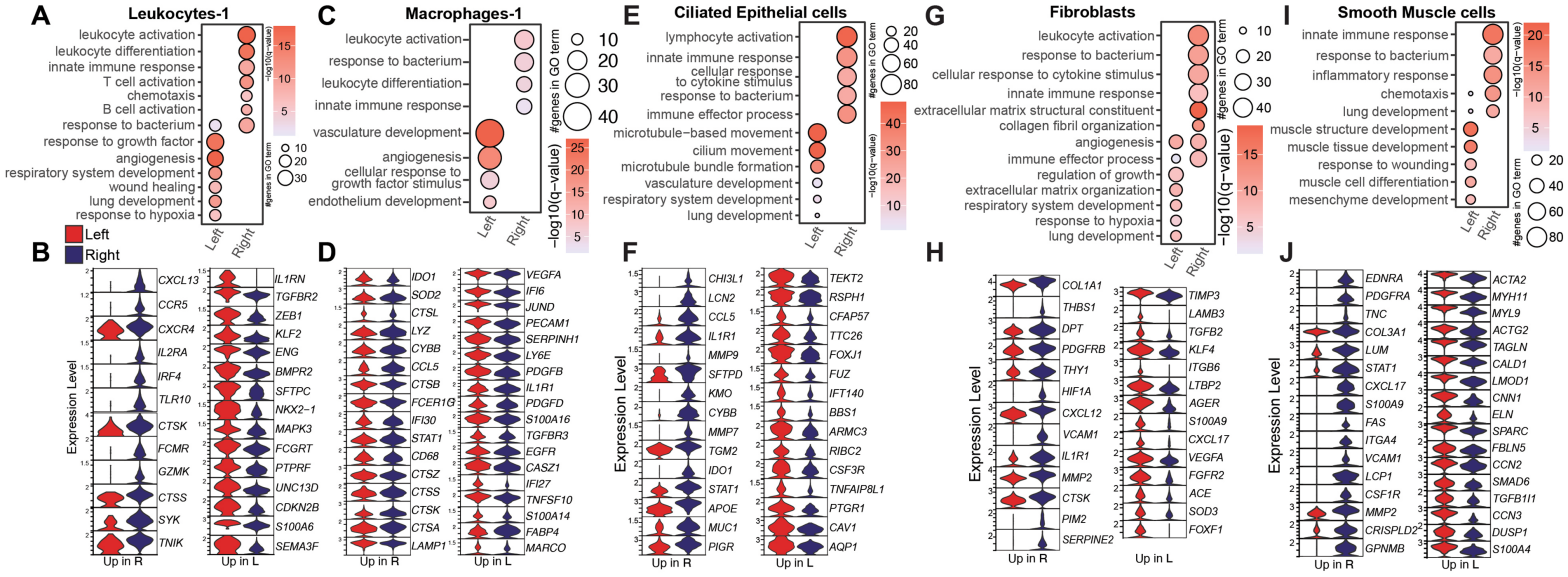
Immune and structural cells are more inflammatory in the right lung. Bubble plots representing Gene Ontology (GO) terms and violin plots depicting differentially expressed genes (DEG) between the right and left lungs from young animals in **(A, B)** leukocytes-1, **(C, D)** macrophages-1, **(E, F)** ciliated epithelial, **(G, H)** fibroblasts, or **(I, J)** smooth muscle cells. For bubble plots, the size of the bubble indicates the number of genes that enriched to that GO term and color indicates the significance compared to aged samples.

We detected the largest number of DEGs when comparing the right (infected) and left (infected) lung sections within leukocyte-1 and macrophage-1 clusters. The leukocyte-1 clusters in the right lungs displayed a transcriptional profile consistent with heightened immune activation and differentiation, with enrichment to gene ontology (GO) terms related to leukocyte activation and bacterial responses (Figure 2A). This was driven by elevated expression of chemokines, cytokine receptors, and transcriptional regulators (*CXCL, CCR, IL2, IRF4, GZMK, SYK*) (Figure 2B). In contrast, genes upregulated in leukocytes-1 from the left lung enriched to “wound healing” and “lung development” pathways (*TGFBR*, *KLF2, ENG, BMPR2, PTPRF*) (Figures 2A,B). A similar dichotomy was observed in Leukocytes-2, where granuloma-associated cells showed increased transcription of immune effector genes tied to T cell signaling and humoral responses (*IGKC, JCHAIN, LCK, ITK, CCL*, *CXCR* genes*, IL2RG, LTB*) whereas the leukocyte-2 cluster in the left lung expressed genes regulating responses to growth factor and tissue homeostasis (*KLF4, ACE, FOXF1, SMAD6*) (Supplementary Figures S3A,B). Genes upregulated in macrophage-1 subset from the right lung were also indicative of persistent innate immune activation (*CCL5, IFI30, STAT1*) and antigen processing (*IDO1, SOD2, CTS* genes*, LYZ, LAMP1*) (Figures 2C,D). Conversely, genes upregulated in the macrophage-1 subset from the left lung enriched for angiogenesis and barrier function (*VEGFA, SERPINH1, PDGFB, PDGFD, TGFBR3, EGFR*) (Figures 2C,D).

**Figure 3:**
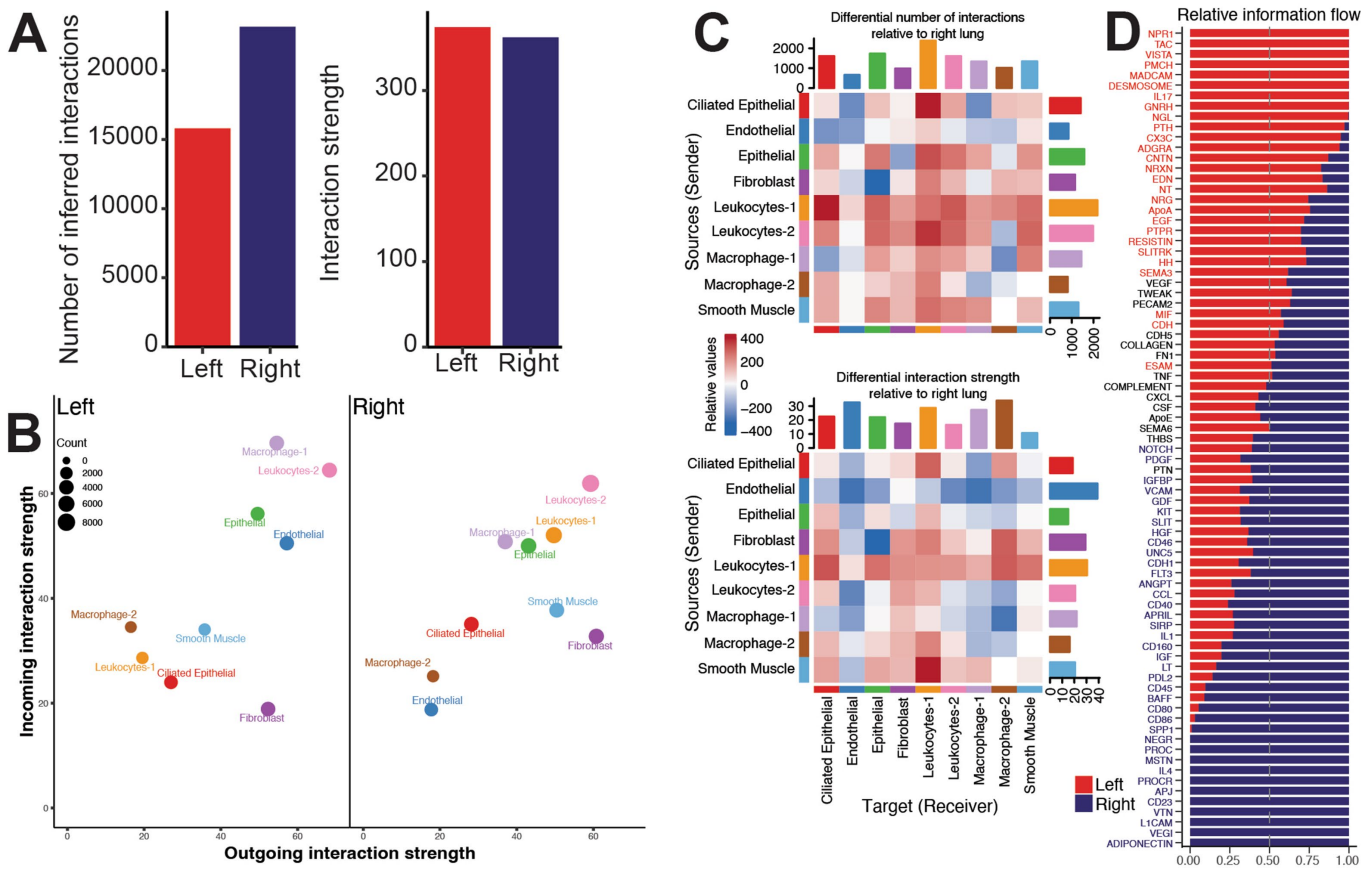
Granuloma-associated macrophages are engaging in host defense while non-granuloma macrophages enrich to repair and growth processes. **(A)** Bar plot of the inferred number (on left) and strength (on right) of ligand-receptor pair interactions in the left and right lung tissue of young animals. **(B)** Scatter plot of incoming and outgoing interaction strength for the indicated cluster. Size of the bubble indicates the number of interactions for the indicated cluster. **(C)** Heatmap demonstrating the differential number of interactions (on top) and the differential interaction strength (on bottom) of the indicated cell clusters relative to the right versus left lungs. Red and blue colors indicate increased or decreased interactions, respectively, in the right tissue relative to left tissue in the young animals. Bar plot on top indicates the sum of interactions sent by the indicated cluster. Bar plot on side indicates the sum of interactions received by the indicated cluster. **(D)** Bar plot of the top signaling pathways ranked by relative information flow (aggregate probability of communication) in the left and right lung tissue. Bars predominantly red denote pathways dominant in left lung whereas bars predominantly blue highlight pathways dominant in right lung.

Non-immune cells in the right lung displayed a distinctly pro-inflammatory and antimicrobial transcriptional program, whereas those in the left lung were enriched for developmental and regulatory transcriptional programs (Figures 2E-J). For example, ciliated epithelial cells from the right lung showed elevated expression of chemokines and cytokine-related genes, including *CCL5, IL1R1, CYBB,* and *STAT1* whereas those in the left lung upregulated expression of genes associated with cilium movement and lung development (*RSPH1, CFAP57, TTC26, FOXJ1, FUZ, CSF3R*) (Figures 2E,F). These compartment-specific patterns were consistently observed across additional epithelial (Supplementary Figures S3C,D) and endothelial cell subsets (Supplementary Figures S3E,F). A similar pattern emerged when comparing stromal cells (Figures 3G-J). Fibroblasts and smooth muscle cells in the right lung upregulated transcripts linked to innate immune activation and antibacterial responses (*THBS1, DPT, IL1R1, HIF1A, STAT1, S100A9, CXCL17*) (Figures 3G-J). In contrast, fibroblasts form the left lung expressed higher levels of genes important for respiratory system development (*TIMP3, LAMB3, TGFB2, VEGFA, FGFR2*), while smooth muscle cells displayed signatures of muscle cell differentiation (*ACTA2, MYH11, MYL9, ACTG2, CNN1*) (Figures 3G-J). Overall, findings among non-immune cells highlight pro-inflammatory and antimicrobial gene programs predominantly in the right lung, while those in the left lung were enriched for tissue homeostasis signatures.

### 3.3 Reduced endothelial signaling and intensified immune signaling within MAH granulomas

We next investigated how divergent cellular activation versus repair programs in the right and left lung reshape intercellular communication networks. Using CellChat (Jin et al., 2021) to infer changes in cell–cell communication networks from the Visium spatial transcriptomics data, we mapped ligand-receptor signaling to define how stromal and immune populations coordinate these distinct responses. Across the 9 identified cell populations, the right lung exhibited a higher number of total ligand-receptor interactions compared to the left lung, whereas overall interaction strength remained comparable between right and left lung compartments (Figure 3A), suggesting increased cellular communications in the right lung.

Both outgoing and incoming signals associated with endothelial cells were markedly stronger in the left lung compared to the right side (Figures 3B,C). In contrast, the leukocyte-1 cluster exhibited significantly stronger outgoing and incoming signals to and from nearly all other populations in the right lung compared to the left lung, underscoring a dominant role for leukocyte signaling within MAH-associated granulomas (Figures 3B,C). Consistent with this observation, fibroblasts, leukocyte-1, and ciliated epithelial cell clusters showed stronger communication with macrophage-2 in the right compared to the left lung, whereas signals originating from endothelial cells, leukocyte-2, and macrophage-1 were stronger in the left lung (Figures 3B,C). Together, these findings suggest that a shift towards immune effector-mediated signaling is important for granuloma formation and/or maintenance.

We next compared the relative information flow for biological signaling pathways that are altered between conditions (Figure 3D). Signaling pathways that were more robust in the left lung were involved in tissue development (PMCH, NGL, EDN, NT, NRG, FGF, SLITRK, HH), cell adhesion (Desmosome, CNTN, NRXN, CDH, ESAM), immune signaling (NPR1, VISTA, MADCAM, IL17, CXC3, PTPR, RESISTIN), and immune cell migration (SEMA3, MIF). Signaling pathways in the right lung were also involved in tissue growth and cell development (NOTCH, PDGF, IGFBP, GDF, KIT, HGF, FLT3, IGF, NEGR, L1CAM), cell migration/survival (SLIT, UNC5, ANGPT), barrier function (VCAM, CDH1, VTN), and inflammation (CD46, CCL, CD40, SIRP, IL1, CD160, LT, CD45, CD80, CD86). Interestingly, some of the signaling pathways in the right lung suggested involvement of B cells (BAFF, CD23), Th2 response (IL4), and anti-inflammatory regulation (PDL2, PROC, VEGI, Adiponectin) (Figure 3D).

Collagen, FN1, MIF, Complement, THBS, CXCL, VEGF, ApoE, and CSF signaling pathways were predicted to be robust in both the left and right lungs (Supplementary Figure S4A). Overall signaling via TWEAK, EGF, PECAM2, EPHB, FLRT, and NRG, which are all important for tissue development, repair, and growth were increased in the left lungs. Pro-inflammatory pathways were overall increased in the right lung indicated by upregulation of CCL, CD40, APRIL, SEMA6, and SPP1 signaling (Supplementary Figure S4A). These signaling patterns are important for immune cell migration (CCL) (Hughes and Nibbs, 2018), co-stimulation (CD40) (Elgueta et al., 2009), B cell survival (APRIL) (Bossen and Schneider, 2006), and tissue maintenance (SEMA6) (Mauti et al., 2007; Toyofuku et al., 2004), as well as cell migration (SPP1) (Poole et al., 2013; Zhao et al., 2024). Fibroblasts, leukocytes-1, macrophage-2, and smooth muscle cells from the right lung all have increased signaling through these pathways. Conversely, endothelial cells from the right lungs have drastically reduced signaling (Supplementary Figure S4A). Network signaling analysis highlights the loss of PECAM2 signaling from endothelial cells to leukocytes-2 and macrophage-1 in favor of towards leukocytes-1 in the right lung (Supplementary Figure S4B). CD40 signaling from leukocytes-1 to all other cell types was greater in the right side (Supplementary Figure S4C). CCL signaling from fibroblasts, leukocytes-1, and smooth muscle cells was also increased in the right lung in line with increased recruitment of immune cells to the site of infection (Supplementary Figure S4D). The most dramatic change was observed in the shift in SPP1 signaling from fibroblasts in the left side to macrophage-2 in the right side (Supplementary Figure S4E).

### 3.4 Hypoxic environments within Mtb-induced granulomas are optimal for mycobacterial growth

Recently, McCaffrey, Delmastro and colleagues have reported distinct zones within granulomas of Mtb infected cynomolgus macaques (McCaffrey et al., 2025). Using a multimodal approach that incorporates spatial transcriptomics, the study showed that the inner caseous necrotic region of the granulomas are surrounded by a hypoxic environment containing macrophages and neutrophils. These peri-necrotic macrophages highly express GLUT1, fibronectin, CD11c, and CD68. Interestingly, T cells are excluded from this region of the granuloma. The hypoxic area also has the highest bacterial burden compared to other regions. The outer myeloid zone surrounding the hypoxic area displayed increased IDO1 expression, which converts tryptophan into kynurenine and has immunosuppressive effects. Despite the potential immunosuppression, macrophages and T cells in this zone exhibited immune activity via reactive oxygen species production, interferon signaling, and epigenetic changes. Finally, lymphocytes dominated the outermost zone. These data suggest that Mtb are able to evade the immune response in hypoxic regions of the granuloma. We utilized the data set obtained from NHP6 granulomas to compare against MAH-induced transcriptional signatures described above in this study.

### 3.5 Distinct macrophage subset dynamics and immune signatures between MAH and Mtb infections

**Figure 4:**
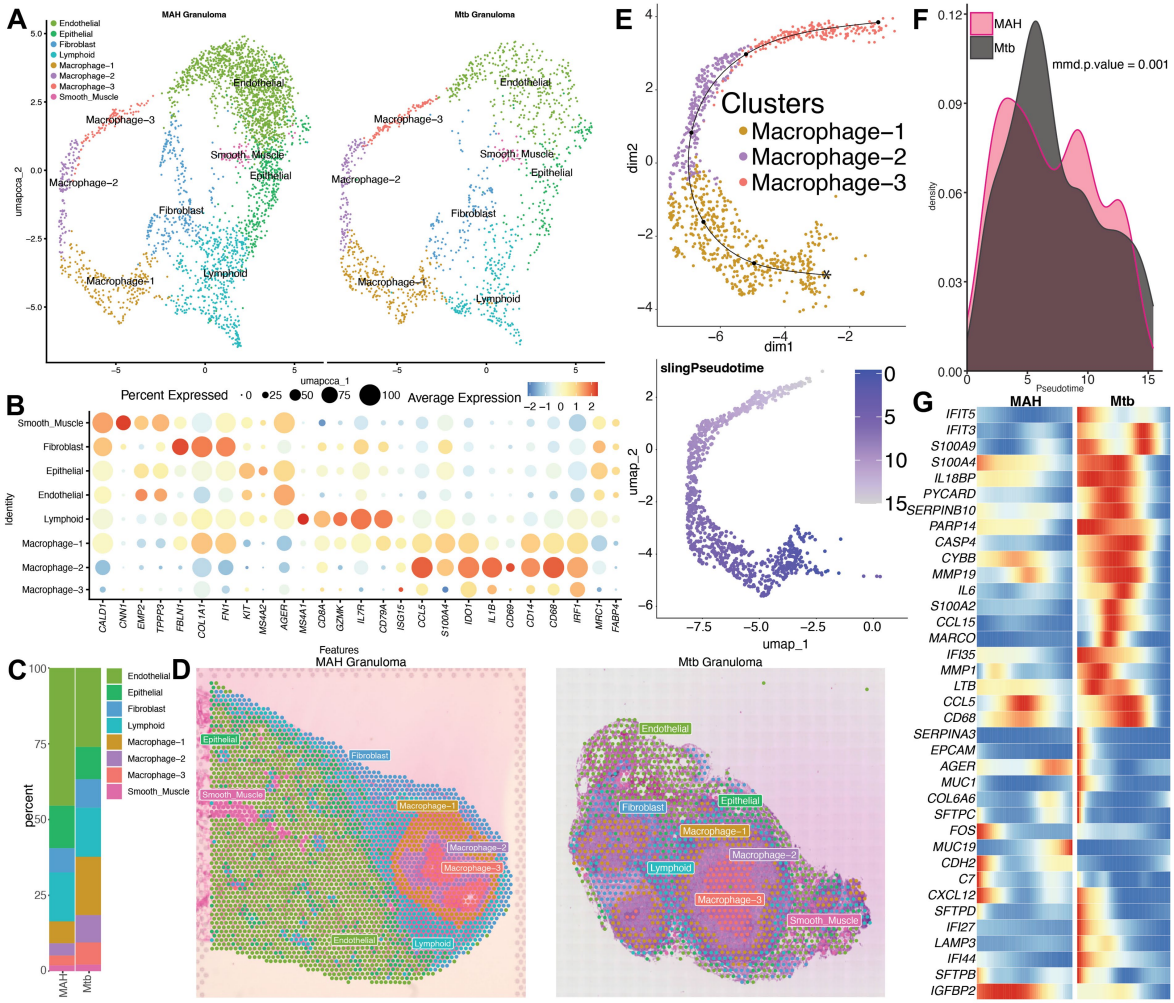
Macrophages of MAH and *Mtb* infected lungs originate in the granulomatous center. **(A)** UMAP of 4,554 spots from granulomatous regions of MAH vs. *Mtb* infected animals. **(B)** Bubble plot of key marker genes used to identify cell populations in panel A. The size of the bubble denotes the percent of cells expressing the marker and color denotes the average expression level of the marker. **(C)** Stacked bar plot of the percent of each cell cluster from the total cells in MAH and *Mtb* infected animals. **(D)** Representative image of H&E-stained MAH and *Mtb* infected lung tissue sections overlayed with cluster identity spots. **(E)** Trajectory analysis of macrophage subsets 1–3 showing the unique clusters (top) and the pseudo time analysis (bottom). Purple represents the origin, and grey represents the terminal point. **(F)** Progression plot showing cell density across pseudotime. **(G)** Differential gene expression heatmap between MAH and *Mtb* tissue across pseudotime.

Comparative analysis of MAH granulomas from this current study and those recently characterized in Mtb-infected cynomolgus macaques (McCaffrey et al., 2025) identified 8 transcriptionally distinct cellular clusters spanning key stromal, immune, and parenchymal populations. Specifically, we identified smooth muscle cells (*CALD1, CNN1*), endothelial cells (*CALD1-, CNN1-, EMP2, TPPP3*), fibroblasts (*FBLN1, COL1A1*), epithelial cells (*KIT, MS1A2*), lymphocytes (*MS4A1, CD8A, GZMK, IL7R, CD79A*), and 3 distinct macrophage subsets (Figure 4A). The ability to resolve a third macrophage subset is likely due to the increased number of cells within these clusters upon merging of the datasets. Macrophage subset 1 demonstrated lower expression of genes that were highly expressed in Macrophage subset 2 (*CCL5, S100A4, IDO1, IL1B, CD69, CD14, CD68, IRF1*) but selectively upregulated *FN1* and *COL1A1*, whereas Macrophage subset 3 was delineated by high *ISG15, CD68,* and *IDO1* expression (Figures 4A,B). Quantitative profiling highlighted a significant decrease in macrophage populations in MAH granulomas compared to Mtb, offset by a marked increase in endothelial cell abundance (Figure 4C). However, this difference in cellular composition is likely due to a tissue sampling bias, with MAH slides exhibiting greater perilesional tissue inclusion in contrast to the more granuloma-restricted sampling of Mtb lesions (Figure 4D).

Trajectory analysis of the macrophage subsets reveals a clear transition from a newly recruited macrophage-1 subset towards the early granuloma-internalized macrophage-2 subset and finally to the macrophage-3 subset, which likely represents the oldest granuloma-associated macrophages (Figure 4E). This aligns with the distance of each cluster to the caseum as well, suggesting that the caseating environment impacts the macrophage phenotype. Importantly, gene expression dynamics observed across pseudotime, spanning these subsets, delineate a clear shift from a pro-inflammatory (*FOSB, C3, S100A16, IRF7, IFI6*) to an anti-inflammatory (*SFRP5, MUC19, SERPINA7, ANGPTL3*) macrophage phenotype (Supplementary Figure S5A), underscoring a potential temporal evolution of macrophage function during granuloma maturation. The progression plot analysis shows a differential macrophage cell density in the two granulomas with a unique peak at pseudotime 5 (transition between macrophage-1 and -2 subsets) with Mtb but a multimodal distribution with MAH (Figure 4F). DEG analysis reveals that macrophages from MAH tissue have lower expression of inflammatory genes (*IFIT5, IFIT3, S100A9, IL6, CCL5, CD68*) compared to Mtb macrophages throughout the pseudotime (Figure 4G). Macrophages from MAH tissue more highly express genes important for cell proliferation (*FOS*), cell–cell adhesion and communication (*CDH2*), and tissue repair (*IGFBP2*) during early stages. Several genes were more prominent in MAH tissue that are responsible for lung homeostasis (*AGER*), maintaining tissue architecture (*COL6A6*), surfactant production (*SFTPC*), mucus production (*MUC19*), and cell adhesion (*CDH2*) during late pseudotime (Figure 4G). These results suggest that macrophages from MAH tissue have a pro-resolution phenotype compared to those from Mtb tissue.

**Figure 5:**
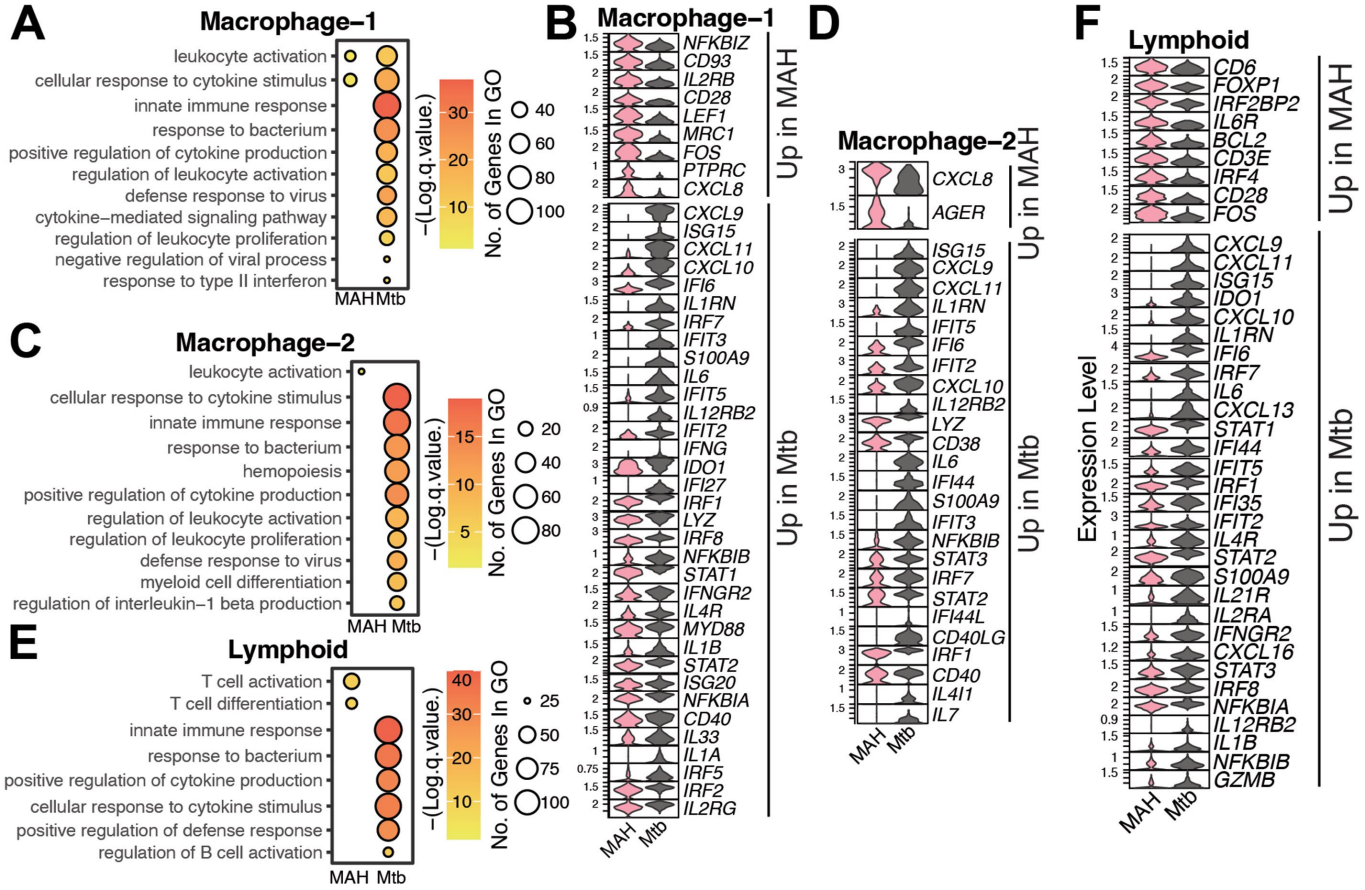
Immune cells in *Mtb*-infected lungs are hyperinflammatory compared to MAH-infected lungs. Bubble plots representing Gene Ontology (GO) terms and violin plots depicting differentially expressed genes (DEG) between the MAH and *Mtb* infected lungs from animals in the **(A,B)** macrophage-1, **(C,D)** macrophage-2, or **(E,F)** lymphoid clusters. For bubble plots, the size of the bubble indicates the number of genes that enriched to that GO term and color indicates the significance compared to aged samples.

Macrophage subsets 1 and 2 from Mtb granulomas expressed higher levels of genes fundamental to antibacterial defense mechanisms, including *S100A9, IL6, LYZ,* and *IL1B,* highlighting an enhanced inflammatory and antimicrobial phenotype in Mtb-driven lesions (Figures 5A–D). Notably, macrophage subset 1 cells in MAH granulomas bore a greater senescent signature, whereas these cells in Mtb granulomas more strongly expressed genes associated with markers of inflammation, apoptosis, and necrosis (Supplementary Figure S5B). For the macrophage subset 2 population, a transcriptional signature of senescence, inflammation, apoptosis, and hypoxia were all significantly elevated in Mtb granulomas (Supplementary Figure S5C). No differentially expressed genes were detected within the Macrophage subset 3.

Lymphoid populations from MAH-infected tissues exhibited heightened expression of key mediators critical for T cell activation and differentiation, including *FOXP1, ZAP70, CD28,* and *IL6R* (Figures 5E,F). In contrast, lymphoid populations from Mtb granulomas demonstrated elevated transcription of genes associated with cytokine and chemokine signaling such as CXCL, interferon stimulated, STAT, interleukin, and alarmin genes (Figures 5E,F). Module scoring analysis further substantiated these divergent immunological states: lymphoid cells in MAH infection were enriched for a senescence-associated transcriptional program, whereas chronic inflammation, apoptosis, hypoxia, and Th1 effector signatures predominated in lymphoid cells from Mtb granulomas (Supplementary Figure S5D). Overall, these findings highlight increased signatures of T cell activation and senescence in MAH granulomas, whereas Mtb granulomas were characterized by heightened inflammatory, apoptotic, and hypoxia responses.

Among structural cells, the expression of key tissue development genes (*MAPK14, GATA6, FOXF1, VEGFA*) were markedly elevated in both endothelial and epithelial cell populations derived from MAH granulomas (Supplementary Figures S6A,B). In contrast, endothelial and epithelial cells from Mtb granulomas demonstrated a pronounced upregulation of inflammatory genes such as *ISG15, IRF1, CXCL9,* and *IL6*, indicating a robust interferon-driven inflammatory environment (Supplementary Figures S6AA–D). Fibroblasts within Mtb granulomas displayed elevated expression of pro-inflammatory genes, recapitulating the broader inflammatory signature, and showed increased levels of tissue remodeling genes *COL1A1* and *FBLN1* (Supplementary Figures S6E,F). Smooth muscle cells from the Mtb cohort also expressed heightened markers of inflammation, including CXCL10, IFI6, and ISG15 (Supplementary Figures S6G,H).

### 3.6 Decreased strength of signaling pathways associated with adaptive immunity, granuloma maintenance, and cell survival in Mtb-induced granulomas

**Figure 6:**
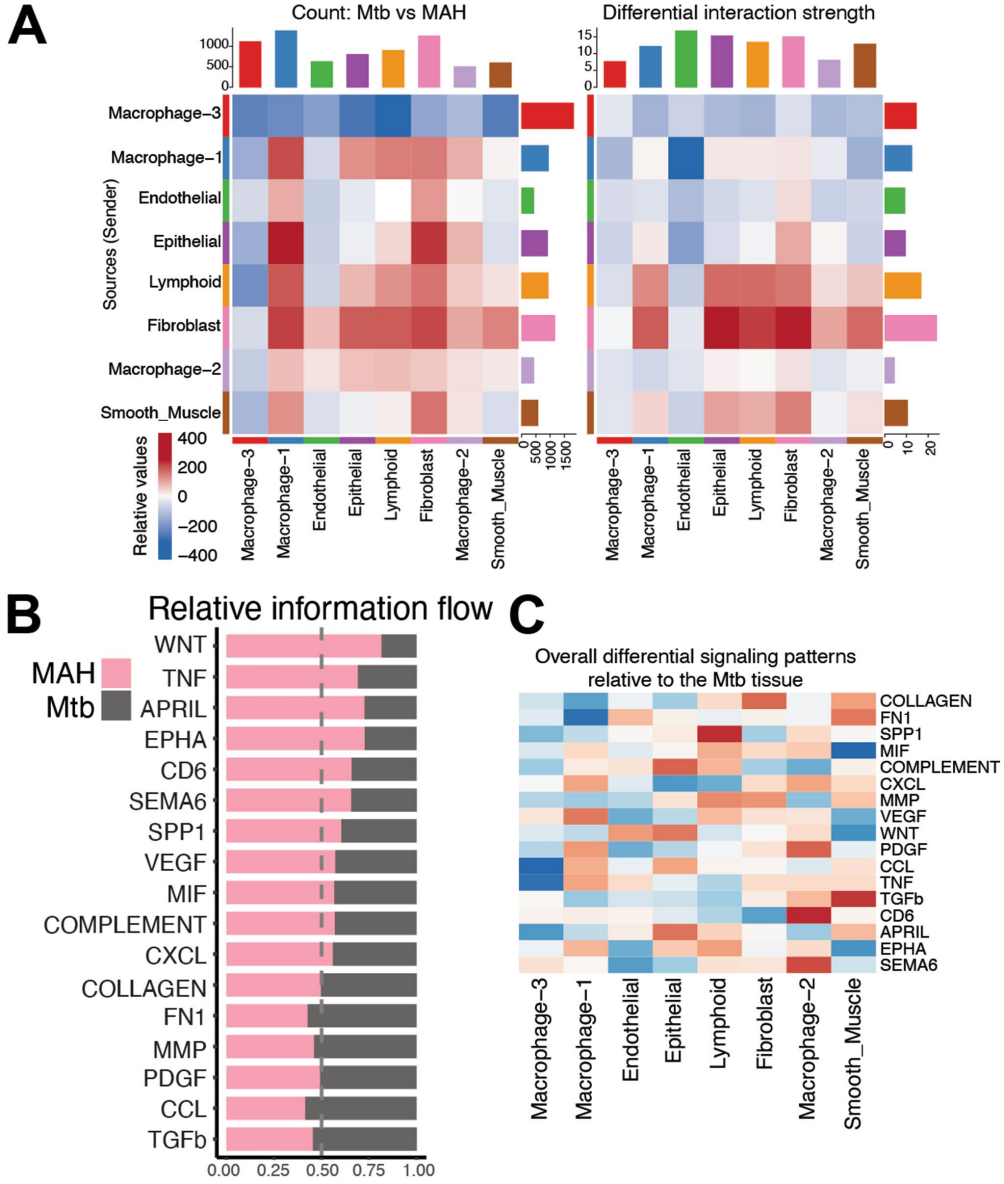
The macrophage-3 subset is not signaling in *Mtb* granulomas. **(A)** Heatmap demonstrating the differential number of interactions (on left) and the differential interaction strength (on right) of the indicated cell clusters between MAH and *Mtb* infected lungs. Red and blue colors indicate increased or decreased interactions, respectively, in the MAH granuloma relative to the *Mtb* granuloma. Bar plot on top indicates the sum of interactions sent by the indicated cluster. Bar plot on side indicates the sum of interactions received by the indicated cluster. **(B)** Bar plot of signaling pathways ranked by relative information flow (aggregate probability of communication) in the MAH and *Mtb* granulomas. Bars predominantly pink denote pathways dominant in MAH granuloma whereas bars predominantly grey highlight pathways dominant in *Mtb* granuloma. **(C)** Differential heatmap of the indicated signaling pathways relative to the *Mtb* granuloma. Blue squares indicate lower signaling in the *Mtb* granuloma compared to the MAH granuloma. Red squares indicate more signaling in the *Mtb* granuloma.

**Figure 7:**
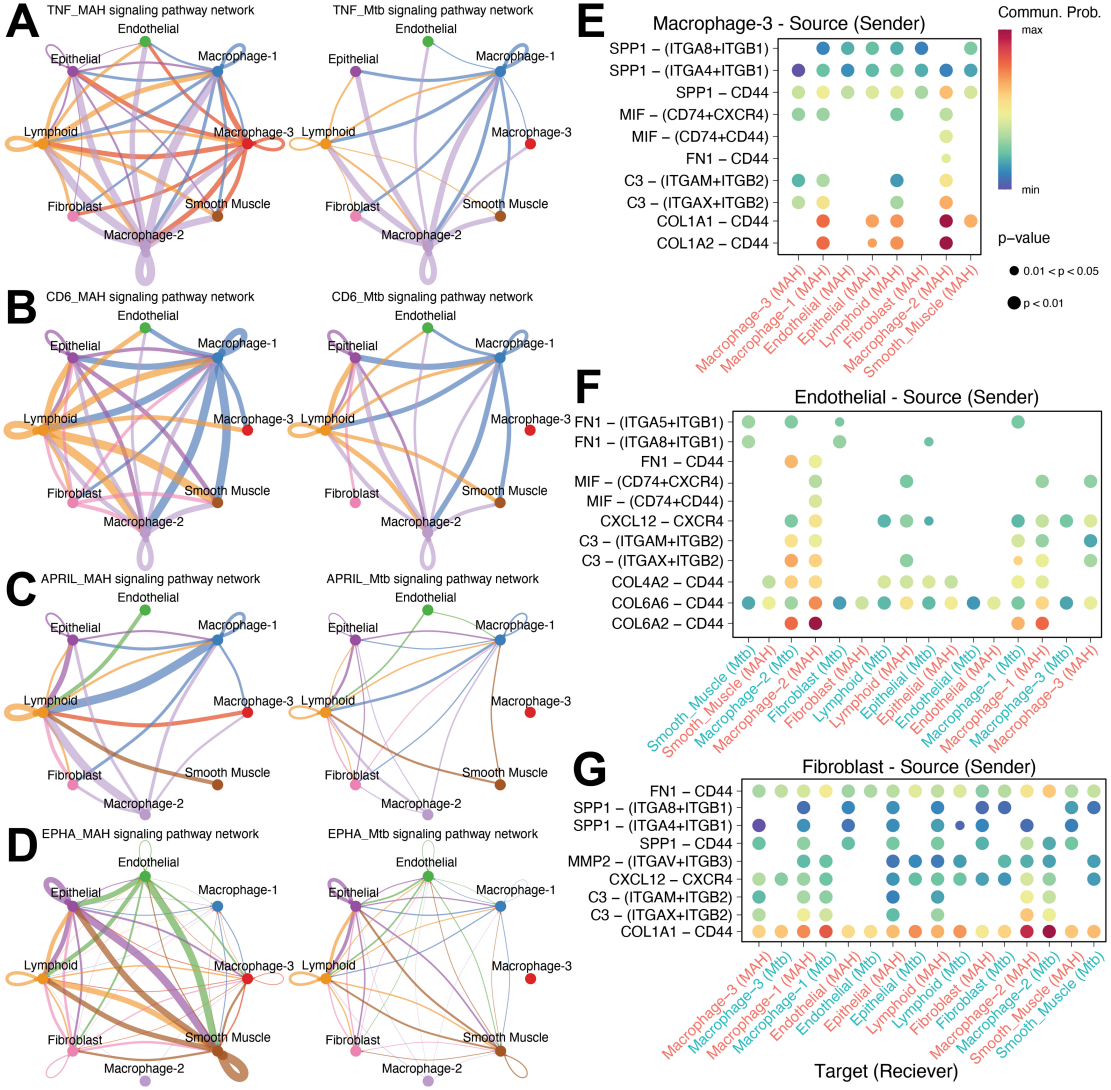
Predicted ligand-receptor pair interactions suggest loss of SPP1 signaling in *Mtb* infected lungs. Network signaling diagrams for the **(A)** TNF, **(B)** CD6, **(C)** APRIL, and **(D)** EPHA signaling pathways among cell subsets in the MAH group (on left) and the *Mtb* group (on right). Nodes represent cell subsets and edge width is proportional to the CellChat communication probability. Bubble plot of differential ligand-receptor pair signaling in MAH or *Mtb* infected lungs from **(E)** the macrophage subset 3, **(F)** endothelial cells, or **(G)** fibroblasts to the other indicated cell type. Color indicates the communication probability and size indicates the *p*-value.

Cell chat analysis of the two granuloma types revealed that macrophage-3, endothelial cells, and fibroblasts had the largest differences in interaction strengths when comparing the MAH and Mtb granulomas (Figure 6A). Fewer incoming and outgoing interactions and diminished strength of interactions was noted in the macrophage-3 subset and endothelial cells in the Mtb group compared to the MAH group. On the other hand, higher number of interactions and increased strength was detected in fibroblasts and macrophage-1 cluster in the Mtb group (Figure 6A). Signaling patterns important for granuloma formation and function such as Collagen, TGF-*β*, MIF, SPP1, FN1, Complement, MMP, CXCL, CCL, PDGF, and VEGF were equally present between the MAH and Mtb granulomas (Figures 6B,C). Interestingly, TNF, WNT, CD6, APRIL, EPHA, and SEMA6 signaling pathways were reduced in Mtb granulomas compared to MAH granulomas (Figures 6B,C). These pathways are important for macrophage and lymphocyte activation as well as cellular migration and tissue remodeling. Specifically, TNF signaling is important for inflammation, immune activation, and granuloma integrity (Clay et al., 2008); WNT signaling helps activate macrophages to repair tissue (Schaale et al., 2013); CD6 signaling activates T cells (Goncalves et al., 2018); APRIL signaling induces B cell maturation and survival (Vincent et al., 2013); and EPHA and SEMA6 signaling causes immune cell movement into the granuloma (Khounlotham et al., 2009). Network signaling analysis highlights the increase of TNF signaling in the lymphoid and macrophage-3 subsets from the MAH compared to Mtb granulomas (Figure 7A). Lymphoid, macrophage-1 and -2, as well as epithelial and fibroblast subsets have more signaling via CD6 in MAH compared to Mtb granulomas (Figure 7B). Increased B cell activation is suggested by enhanced APRIL signaling in MAH granulomas from lymphoid and macrophage subsets (Figure 7C). EPHA signaling is increased in the MAH granuloma predominantly through epithelial, endothelial, and smooth muscle cells (Figure 7D).

Since macrophage subset 3, endothelial cell, and fibroblast signaling was the most changed, we investigated specific ligand-receptor pair interactions in these clusters (Figures 7E-G). Macrophage-3 signaling was only occurring in the MAH infected lungs (Figure 7E). COL1A (collagen signaling pathway) ligands were predicted to participate in most of the signaling to other cell types. C3 (complement), FN1 (fibronectin), MIF (macrophage survival), and SPP1 (immune signaling) interactions were also highly probable. Macrophage-3 to macrophage-2 signaling was predicted to involve predominantly MIF, FN1, and C3 signaling pathways (Figure 7E). Collagen signaling was most strongly predicted to originate from endothelial cells (Figure 7F). Interestingly, signaling through FN1 was predicted from endothelial cells towards smooth muscle, macrophage-2, fibroblasts, epithelial, and macrophage-1 subsets of Mtb infected lungs, but not MAH infected lungs. Conversely, MIF signaling from endothelial cells was predicted towards lymphoid and all macrophage subsets of MAH infected lungs, but not Mtb infected lungs. Endothelial cells did not send C3 signals to macrophage-3 or lymphoid subsets of Mtb infected lungs (Figure 7F). Fibroblast signaling was predicted to mostly involve collagen and FN1 networks (Figure 7G). Most of the predicted fibroblast interactions were similar between MAH and Mtb infected lungs. However, SPP1 signaling through ITGA8 + ITGB1, or ITGA4 + ITGB1, or both in all cell types was significantly reduced in Mtb infected lungs (Figure 7G), thus indicating a defect in tissue repair.

## 4 Discussion

We performed spatial transcriptomic analyses to investigate the structural and immunological state of lung tissue exhibiting MAH granulomatous disease, which is one of the most common clinical manifestations of NTMPD (O'Connell et al., 2012). These complex immune structures are crucial for controlling the spread of mycobacteria. This is accomplished by attracting macrophages and lymphocytes to form a cellular structure around the pathogen. However, if the pathogen is able to subvert the immune response or if the host’s immune system is weakened, granulomatous formation can become a benefit to mycobacteria rather than an effective host defense response (Bold and Ernst, 2009).

Lung tissue biopsies from right side harboring MAH granulomas exhibited an antimicrobial and inflammatory gene signature, while the left lung was in a transcriptional state of repair. The inflammatory signature in the right lung could be due to persistence of MAH antigen (Pan et al., 2017) or lack of resolution of the inflammatory process. Cell chat analysis of granuloma-associated macrophages revealed that they expressed high levels of genes involved in antimicrobial responses and other airway diseases including *CCL5* (Vesosky et al., 2010), *C3* (Lenhart-Pendergrass et al., 2023), *IL-33* (Drake and Kita, 2017), and *MIF* (Das et al., 2013). In addition, as seen with Mtb in humans (Qiu et al., 2024), upregulation of collagen genes and *THBS1* (thrombospondin-1) was a feature of MAH granulomatous tissue. Immune and parenchymal cells in granuloma-rich right lungs demonstrated robust upregulation of genes related to immune activation, chemokine/cytokine signaling, and antimicrobial defense (CXCL, CCR, IL2, IRF4, GZMK). In contrast, corresponding populations in the left lung displayed transcriptional signatures indicative of quiescence, tissue repair, angiogenesis, and lung development (TGFBR, KLF2, VEGFA, FOXF1). These findings align with other models showing that granulomas create a proinflammatory microenvironment (Krueger et al., 2025). The reparative and developmental signatures observed in the non-infected side mirrors findings from studies in post-infection and convalescent lungs (Lucas et al., 2020).

Comparison of the MAH granulomas from rhesus macaques with Mtb granulomas from cynomolgus macaques identified three macrophage subsets with subset 2 and 3 localizing in the center of the granulomas and expressing proinflammatory marker genes. The macrophage subset 1 circled the outside of the granulomatous center. Interestingly, the macrophage-1 subset expresses *FN1*, which is upregulated in lung macrophages during injury and inflammation (Fei et al., 2018). Trajectory analysis showed that the macrophage-1 subset transitions to macrophage-2 and finally to macrophage-3 within the granuloma. DEG analysis showed that macrophages from Mtb tissue were more inflammatory at all stages compared to those from MAH tissue. An in-depth analysis of the ligand-receptor interactions revealed that the macrophage 3 subset is likely to communicate with macrophage subset 2 via complement, MIF, and SPP1 signaling pathways. Inflammatory signaling between macrophage-3, which resides in the necrotic center, and macrophage-2, which surrounds subset 3, align topologically with the lung histology. We propose that the lack of macrophage-3 signaling in Mtb-infected lungs suggests that these cells are dying.

Immune and non-immune cells expressed a heightened inflammatory profile in the Mtb versus the MAH granuloma. DEGs from the MAH tissue enriched to signaling patterns important for adaptive immune activation and maintaining granuloma integrity. Immune cells in granulomatous tissue of MAH infected animals only exhibited T cell or leukocyte activation. In contrast, tissue of Mtb infected animals had a heightened transcriptional response indicative of activated innate immunity but regulated adaptive activation. Mtb is known to skew the immune response away from a Th1 signature (Cao et al., 2019).

Fibroblasts from Mtb-infected lungs had increased signaling, which indicates increased fibrosis (Lee et al., 2019). Several signaling cascades were lacking in Mtb granulomatous tissue such as those involving the Th1-associated molecule TNF (Clay et al., 2008), CD6 (Goncalves et al., 2018), and APRIL (Vincent et al., 2013) which activate the adaptive arm of the immune system. Loss of EPHA and SEMA6 signaling may explain the lack of adaptive immunity activation and worsened granulomatous disease in Mtb infected lungs since they are critical for immune cell migration into the granuloma (Khounlotham et al., 2009) while WNT signaling is important for macrophages to start to repair tissue (Schaale et al., 2013).

Endothelial cells surrounding Mtb granulomas had a higher probability of sending out FN1 signals, which may indicate increased cellular senescence (Kumazaki et al., 1991). Conversely, endothelial cells from MAH granulomas were predicted to send MIF signals which promote macrophage survival (Calandra and Roger, 2003). While fibroblast signaling was overall increased in Mtb-granuloma, SPP1 signaling through ITGA8 + ITGB1 or ITGA4 + ITGB1 was predicted to be lost. The lack of two cell survival signals (MIF and SPP1) may explain why the macrophage-3 subset is transcriptionally unresponsive in Mtb granulomas. Transcriptionally, the major difference between the Mtb versus the MAH granuloma is the excessive inflammation combined with the lack of crucial signaling patterns that would activate adaptive immune cells. The Mtb granuloma was previously shown to be hypoxic (McCaffrey et al., 2025) and the transcriptomic signature suggested it was more hypoxic by module scoring than the MAH granuloma.

In line with our observations, prior studies have reported increased cathepsin gene expression across immune and structural cells (*CTSK*, *CTSS*, etc) in granulomatous tissue. Indeed, Cathepsin K has been used as a biomarker to identify micro-granulomas during Crohn’s disease (Pedica et al., 2009). *CCL5* has been shown to be highly expressed in granulomatous tissue (Castellani et al., 2007) and is crucial for the immune response to mycobacterium (Vesosky et al., 2010). *CCL5* transcripts were also upregulated in macrophages and ciliated epithelial cells from granulomatous tissue. *STAT1* transcripts were also upregulated across cell types, and this has been shown to have increased expression in granulomas during sarcoidosis (Rosenbaum et al., 2010).

This study has some limitations. The first generation of Visium transcriptomics was used to investigate MAH and Mtb granuloma. Future studies can leverage high definition Visium which allows analysis at the single cell levels to achieve greater resolution and insight. The granulomatous region was not normalized between the MAH and Mtb granulomas, preventing direct comparison of cell frequencies. The lung tissue was also collected at different times after infection and came from different macaque species, that may impact the results. Future studies should consider infecting with multiple species in MAC, such as *M. intracellulare* and *M. chimera* to better recapitulate a natural infection. This study is also limited by the lack of validation of differential gene expression via protein-based assays and should be included in future studies. Nevertheless, these data reveal a granuloma-associated macrophage population in both models. Cells in the granulomatous region in the infected right lungs continue to respond to MAH, but those from left lungs were focused on lung repair. Major signaling changes were identified in macrophages, endothelial cells, and fibroblasts. Mtb infected lungs appeared to lose signaling patterns important for adaptive immunity activation and maintaining granuloma integrity. Finally, Mtb granulomas have more FN1 senescent signaling from endothelial cells but lack MIF and SPP1 signaling for survival compared to MAH granulomas.

## Data Availability

The datasets presented in this study can be found in online repositories. The names of the repository/repositories and accession number(s) can be found at: NCBI, accession number PRJNA1247551.
