# Correction: Granulomatous cellular signatures in nontuberculous and tuberculous mycobacterial infections

**DOI:** 10.3389/fmicb.2026.1794732

**Published:** 2026-02-09

**Authors:** Brianna M. Doratt, Ethan G. Napier, Mahdi Eskandarian Boroujeni, Sarah Douglas, Michael H. Davies, Luiz Bermudez, Eliot R. Spindel, Erin F. McCaffrey, Ilhem Messaoudi

**Affiliations:** 1 Department of Microbiology, Immunology, and Molecular Genetics, College of Medicine, University of Kentucky, Lexington, KY, United States; 2 Spatial Immunology Unit, Laboratory of Parasitic Diseases, National Institute of Allergy and Infectious Disease, National Institutes of Health, Bethesda, MD, United States; 3 Division of Neuroscience, Oregon National Primate Research Center, Oregon Health and Science University, Beaverton, OR, United States; 4 Department of Microbiology, College of Sciences, Oregon State University, Corvallis, OR, United States

**Keywords:** aging lung, granuloma, macaque, mycobacterium, visium spatial transcriptomics

Figures 2–6 are cropped at the bottom precluding the reader from seeing the full content. The full figures appear below.

**Figure 2:**
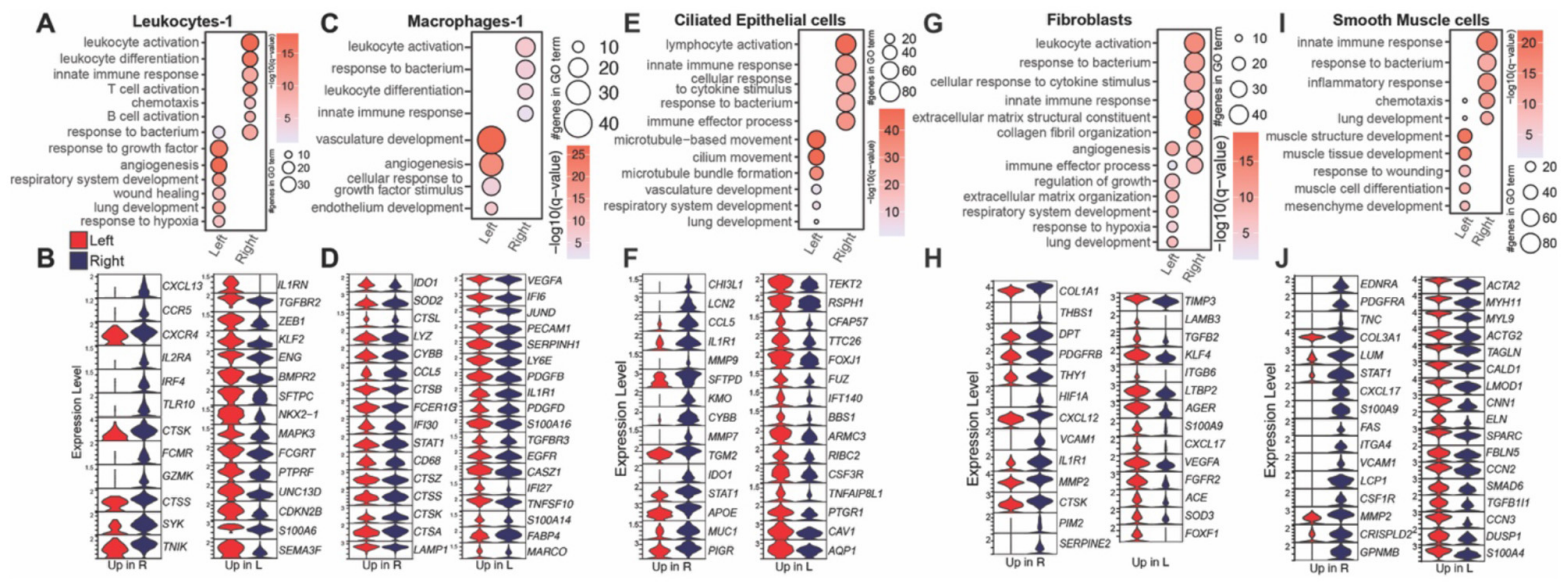
Immune and structural cells are more inflammatory in the right lung. Bubble plots representing Gene Ontology (GO) terms and violin plots depicting differentially expressed genes (DEG) between the right and left lungs from young animals in **(A, B)** leukocytes-1, **(C, D)** macrophages-1, **(E, F)** ciliated epithelial, **(G, H)** fibroblasts, or **(I, J)** smooth muscle cells. For bubble plots, the size of the bubble indicates the number of genes that enriched to that GO term and color indicates the significance compared to aged samples.

**Figure 3:**
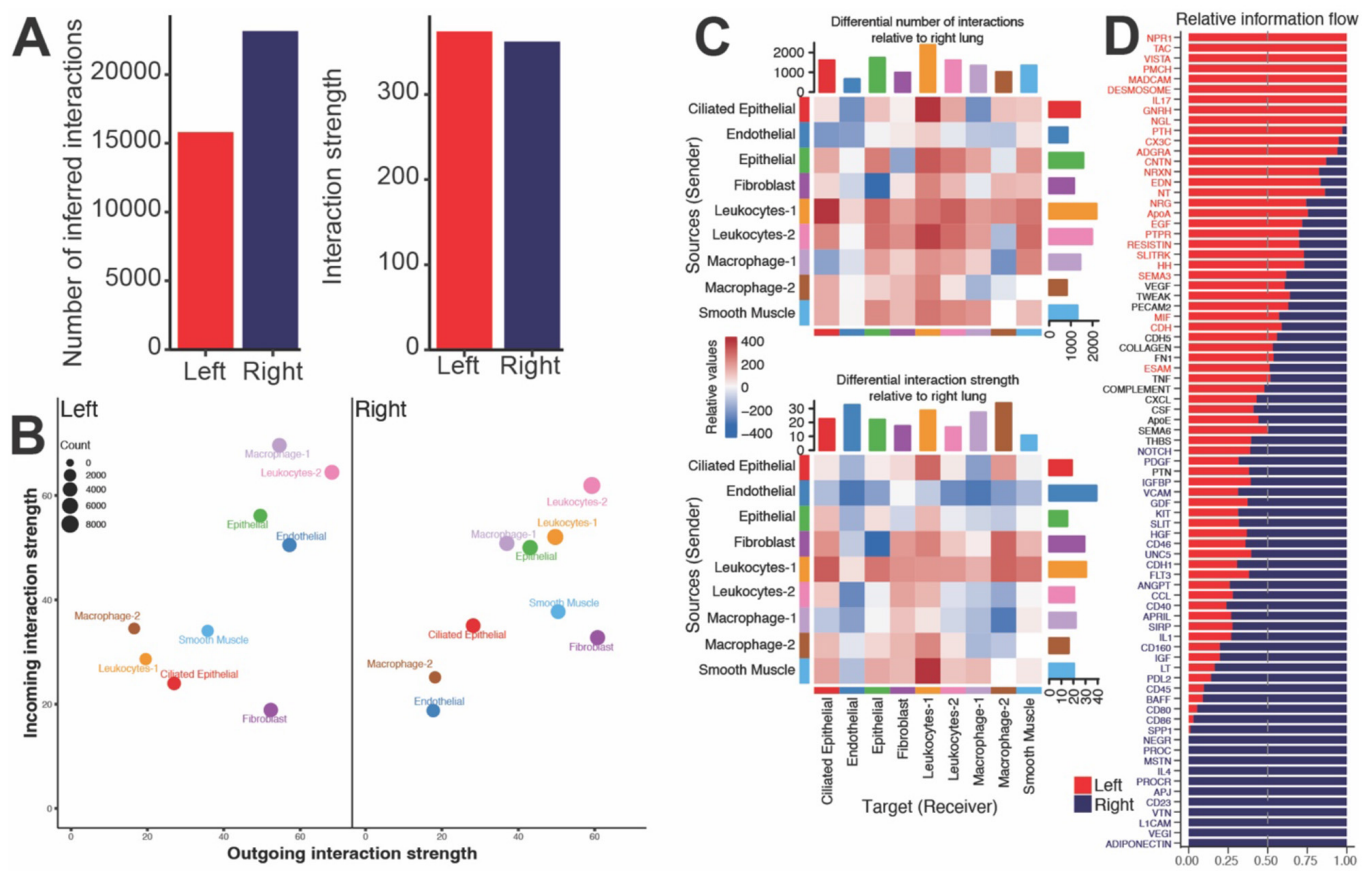
Granuloma-associated macrophages are engaging in host defense while non-granuloma macrophages enrich to repair and growth processes. **(A)** Bar plot of the inferred number (on left) and strength (on right) of ligand-receptor pair interactions in the left and right lung tissue of young animals. **(B)** Scatter plot of incoming and outgoing interaction strength for the indicated cluster. Size of the bubble indicates the number of interactions for the indicated cluster. **(C)** Heatmap demonstrating the differential number of interactions (on top) and the differential interaction strength (on bottom) of the indicated cell clusters relative to the right versus left lungs. Red and blue colors indicate increased or decreased interactions, respectively, in the right tissue relative to left tissue in the young animals. Bar plot on top indicates the sum of interactions sent by the indicated cluster. Bar plot on side indicates the sum of interactions received by the indicated cluster. **(D)** Bar plot of the top signaling pathways ranked by relative information flow (aggregate probability of communication) in the left and right lung tissue. Bars predominantly red denote pathways dominant in left lung whereas bars predominantly blue highlight pathways dominant in right lung.

**Figure 4:**
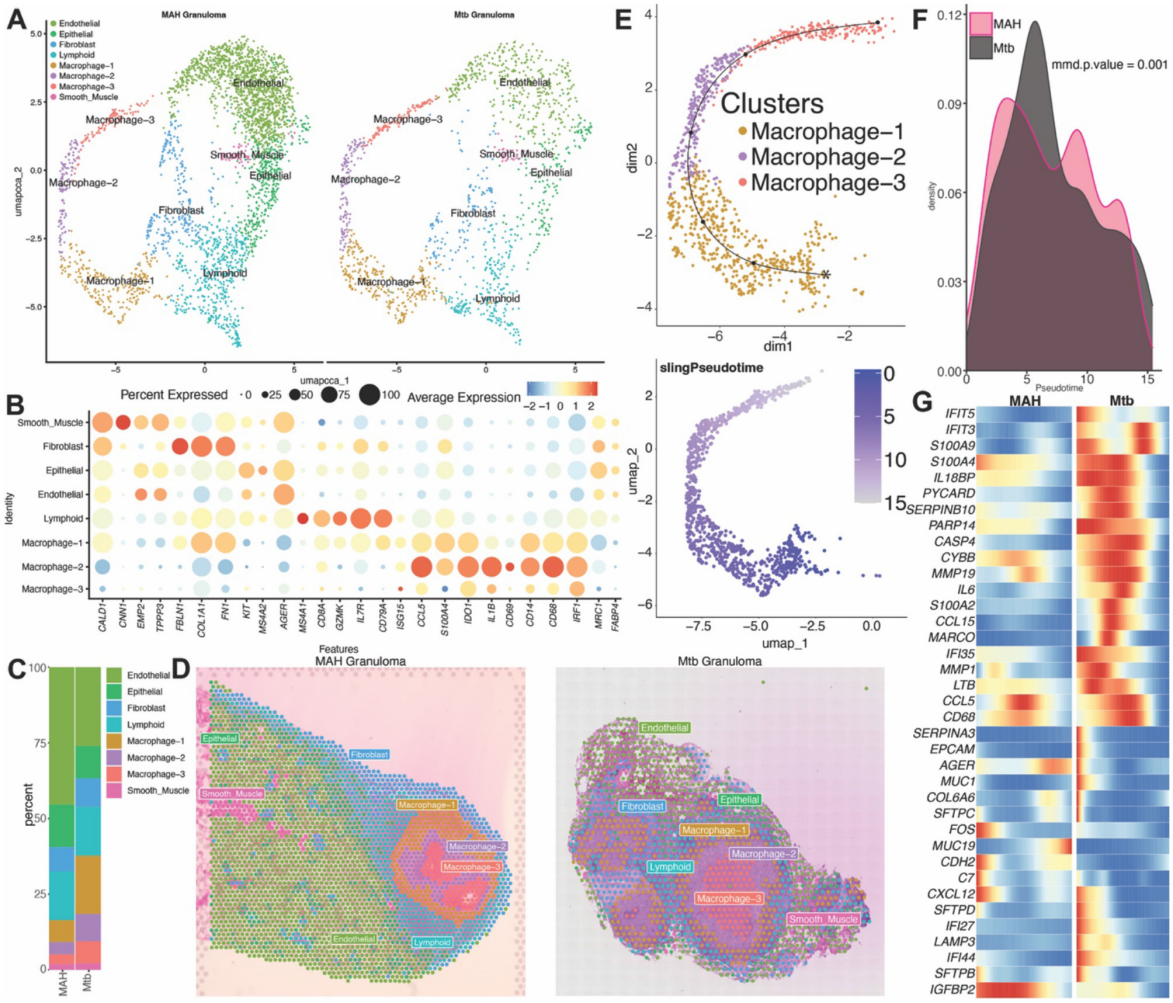
Macrophages of MAH and *Mtb* infected lungs originate in the granulomatous center. **(A)** UMAP of 4,554 spots from granulomatous regions of MAH vs. *Mtb* infected animals. **(B)** Bubble plot of key marker genes used to identify cell populations in panel A. The size of the bubble denotes the percent of cells expressing the marker and color denotes the average expression level of the marker. **(C)** Stacked bar plot of the percent of each cell cluster from the total cells in MAH and *Mtb* infected animals. **(D)** Representative image of H&E-stained MAH and *Mtb* infected lung tissue sections overlayed with cluster identity spots. **(E)** Trajectory analysis of macrophage subsets 1–3 showing the unique clusters (top) and the pseudo time analysis (bottom). Purple represents the origin, and grey represents the terminal point. **(F)** Progression plot showing cell density across pseudotime. **(G)** Differential gene expression heatmap between MAH and *Mtb* tissue across pseudotime.

**Figure 5:**
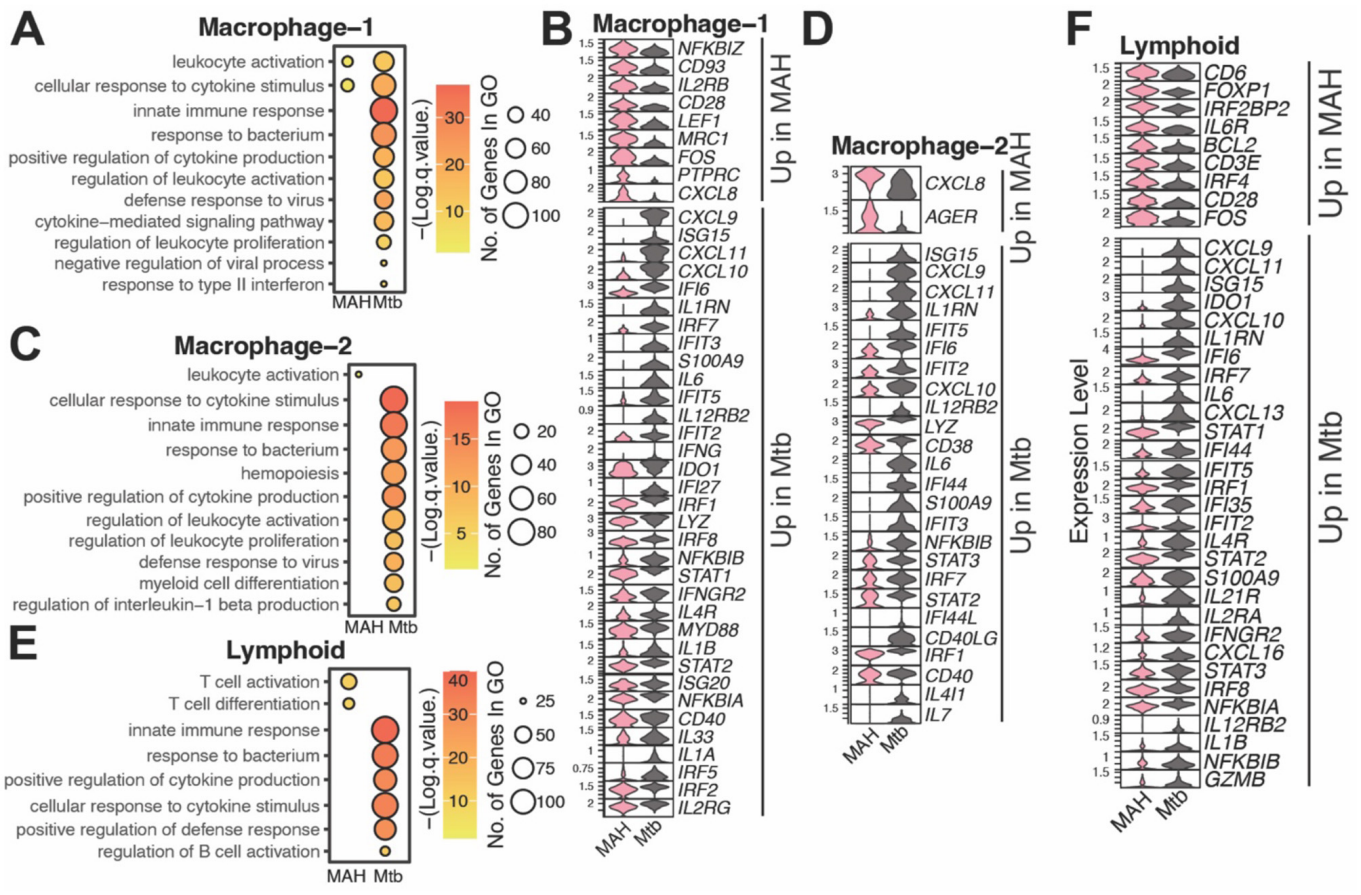
Immune cells in *Mtb*-infected lungs are hyperinflammatory compared to MAH-infected lungs. Bubble plots representing Gene Ontology (GO) terms and violin plots depicting differentially expressed genes (DEG) between the MAH and *Mtb* infected lungs from animals in the **(A, B)** macrophage-1, **(C, D)** macrophage-2, or **(E, F)** lymphoid clusters. For bubble plots, the size of the bubble indicates the number of genes that enriched to that GO term and color indicates the significance compared to aged samples.

**Figure 6:**
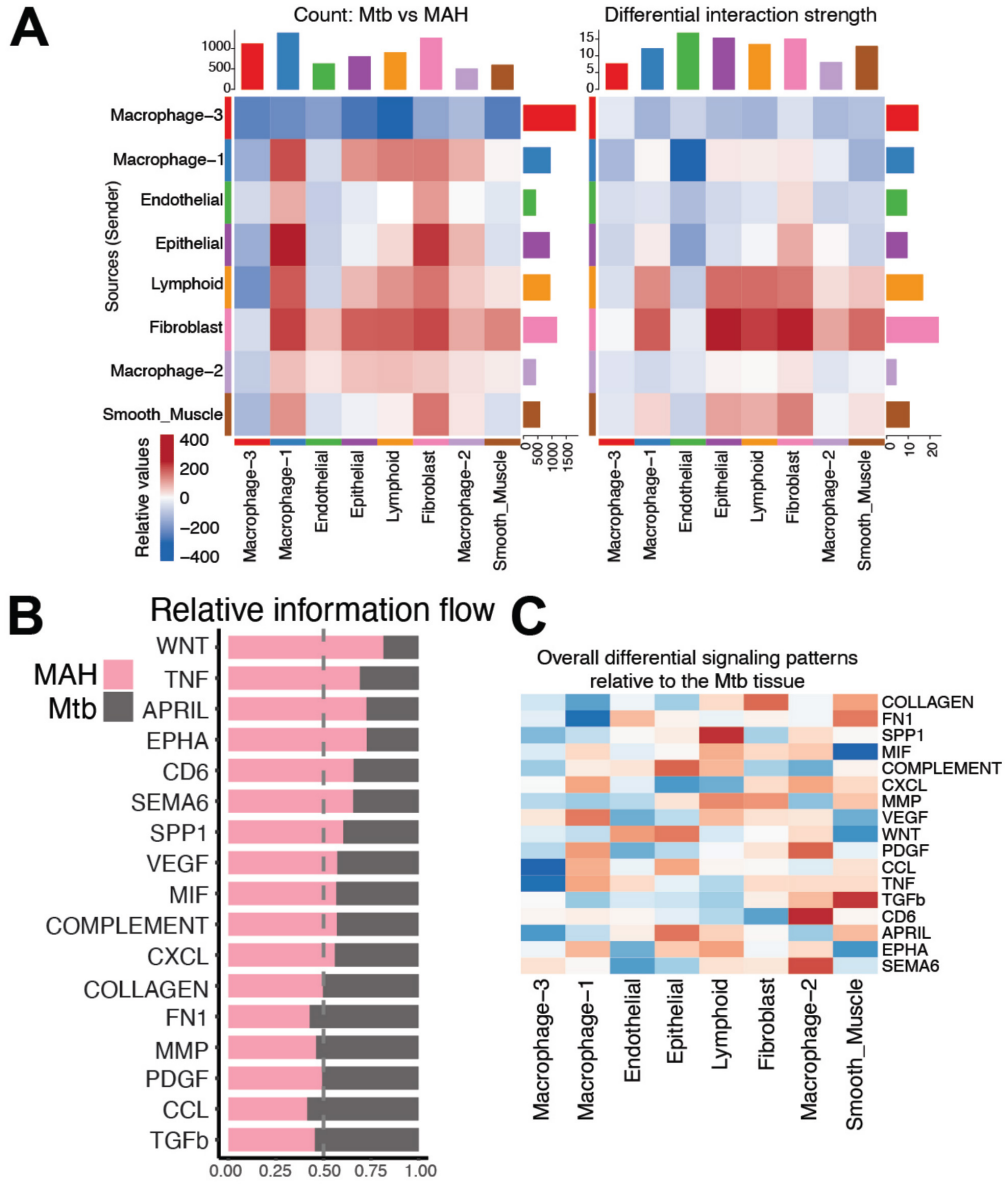
The macrophage-3 subset is not signaling in *Mtb* granulomas. **(A)** Heatmap demonstrating the differential number of interactions (on left) and the differential interaction strength (on right) of the indicated cell clusters between MAH and *Mtb* infected lungs. Red and blue colors indicate increased or decreased interactions, respectively, in the MAH granuloma relative to the *Mtb* granuloma. Bar plot on top indicates the sum of interactions sent by the indicated cluster. Bar plot on side indicates the sum of interactions received by the indicated cluster. **(B)** Bar plot of signaling pathways ranked by relative information flow (aggregate probability of communication) in the MAH and *Mtb* granulomas. Bars predominantly pink denote pathways dominant in MAH granuloma whereas bars predominantly grey highlight pathways dominant in *Mtb* granuloma. **(C)** Differential heatmap of the indicated signaling pathways relative to the *Mtb* granuloma. Blue squares indicate lower signaling in the *Mtb* granuloma compared to the MAH granuloma. Red squares indicate more signaling in the *Mtb* granuloma.

The original version of this article has been updated.

