# Supplementary figures and images for: Granulomatous cellular signatures in nontuberculous and tuberculous mycobacterial infections
Source: Front Microbiol. 2026 Jan 8;16:1741883. doi: 10.3389/fmicb.2025.1741883 (PMC12823825; doi:10.3389/fmicb.2025.1741883)

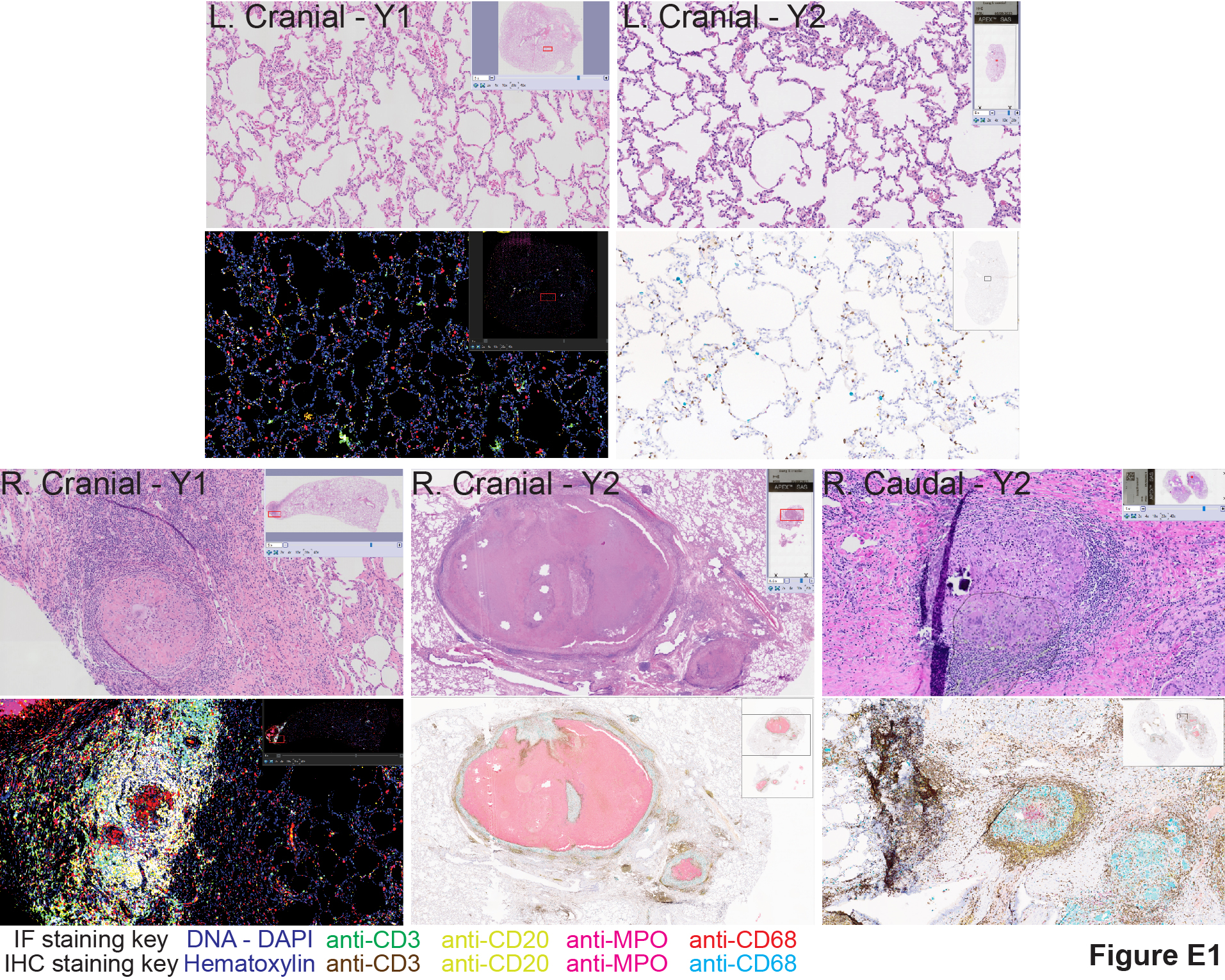

Supplement: Supplementary file 9 [file Image_1.JPEG]

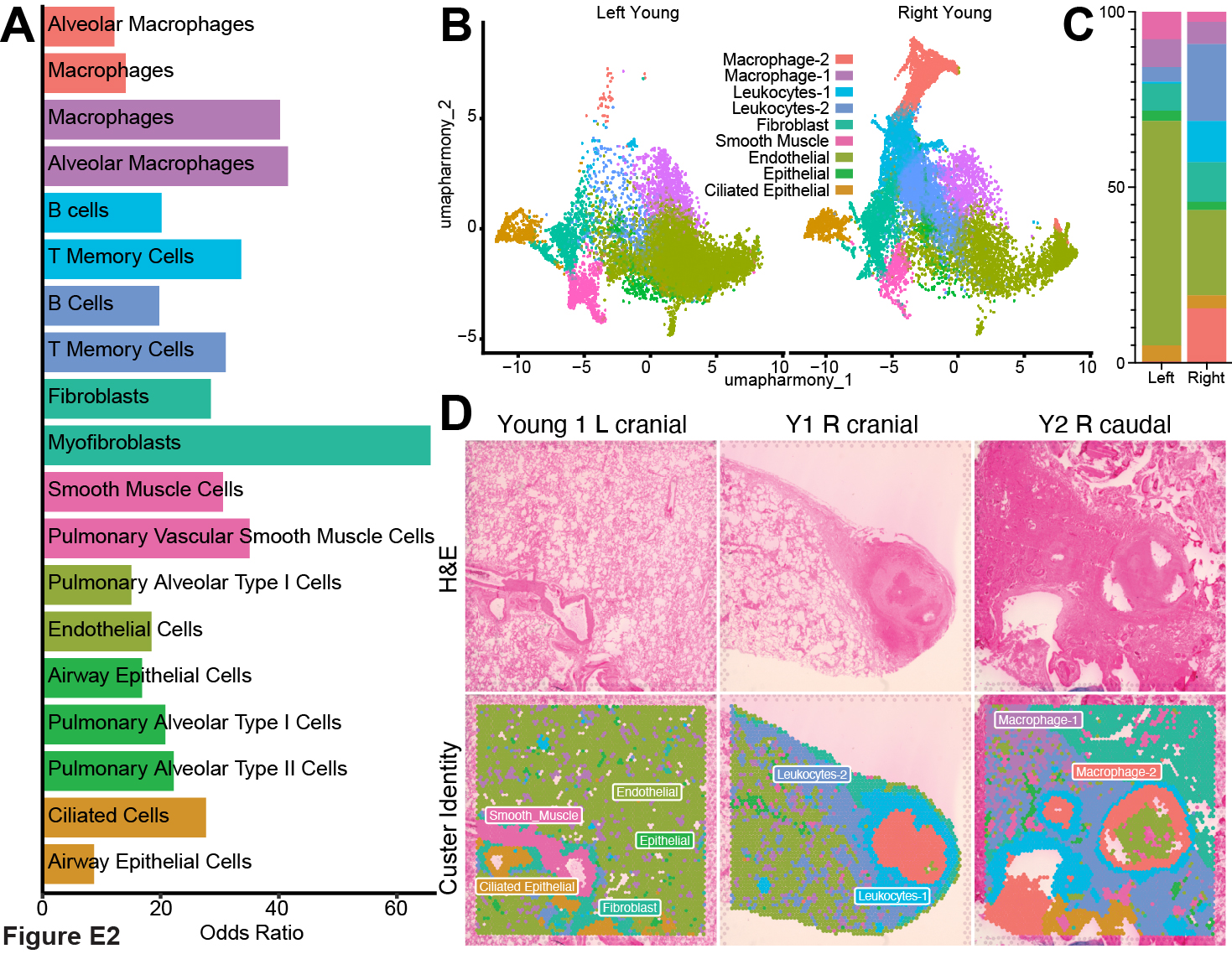

Supplement: Supplementary file 10 [file Image_2.JPEG]

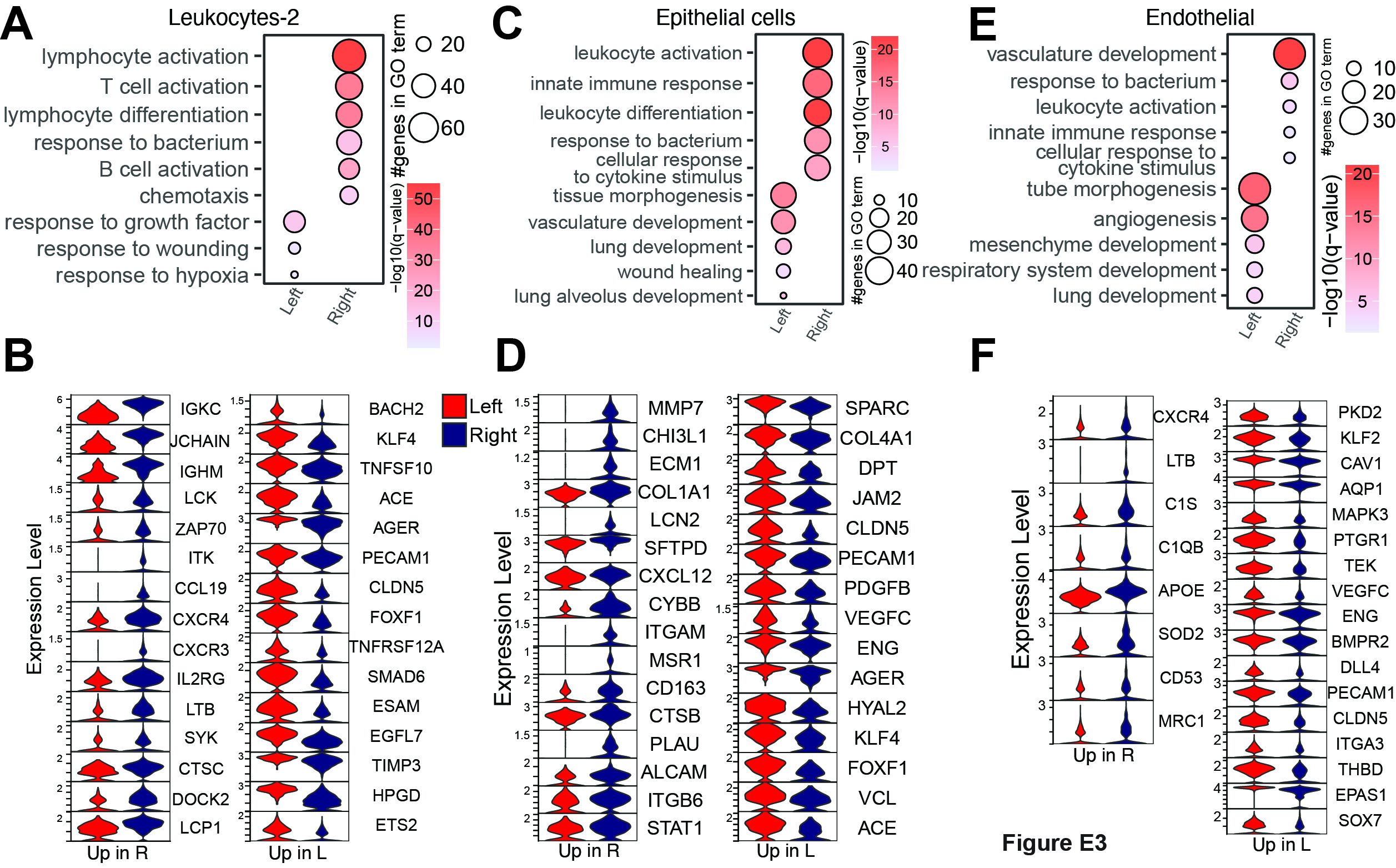

Supplement: Supplementary file 11 [file Image_3.JPEG]

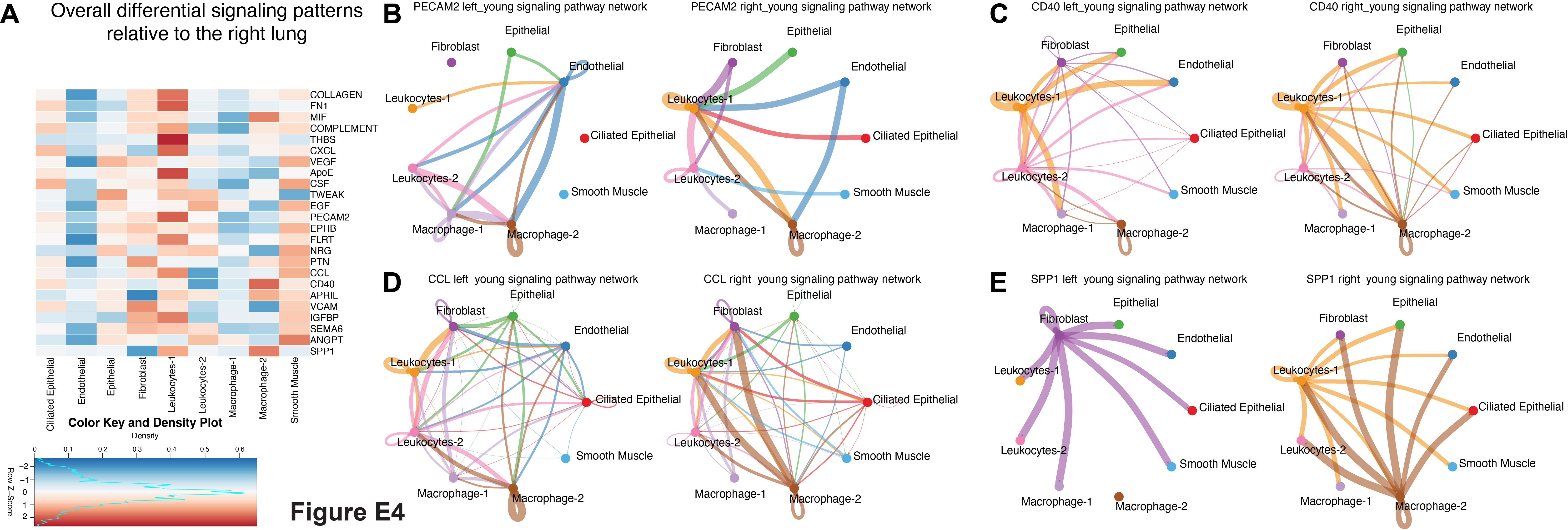

Supplement: Supplementary file 12 [file Image_4.JPEG]

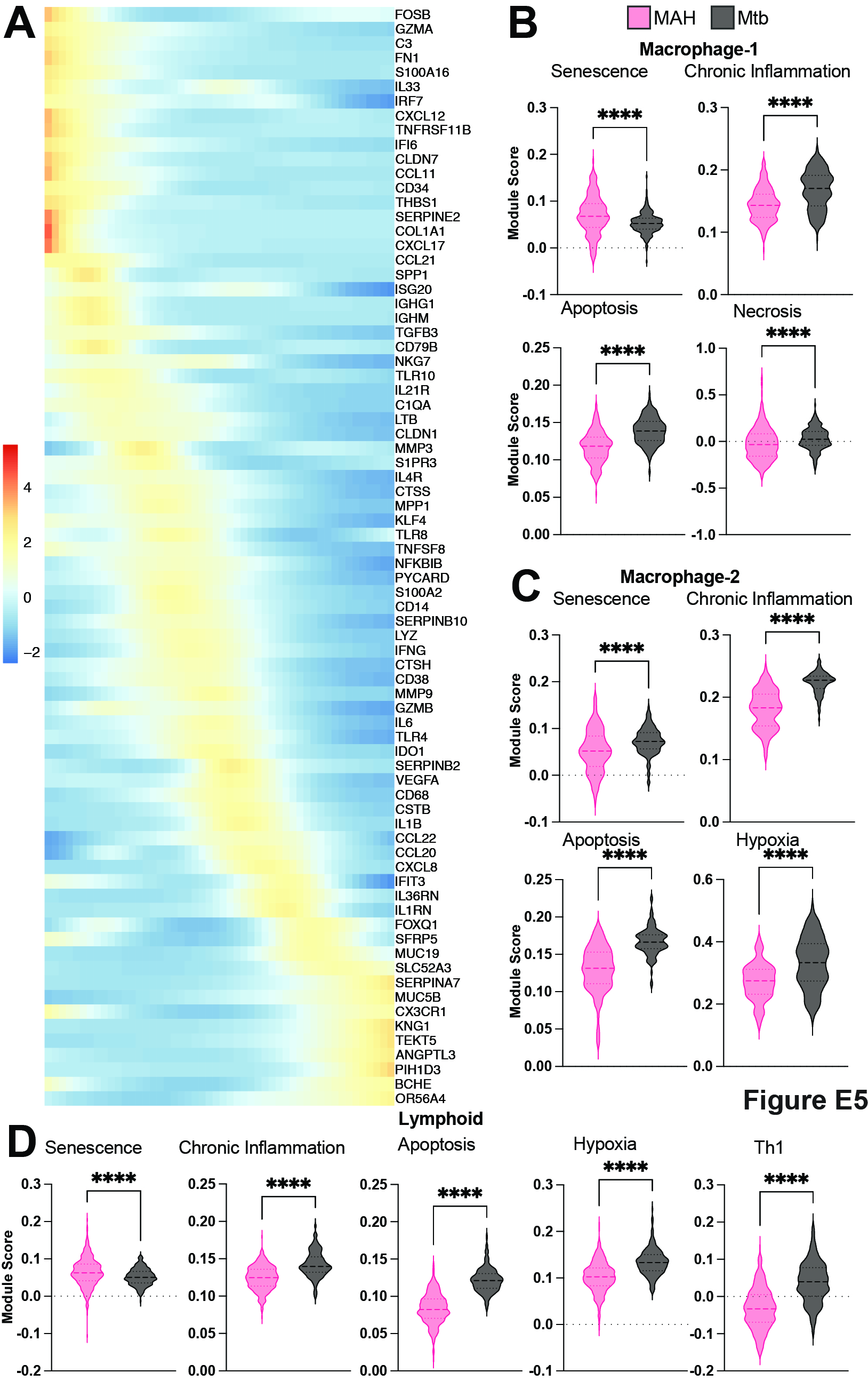

Supplement: Supplementary file 13 [file Image_5.JPEG]

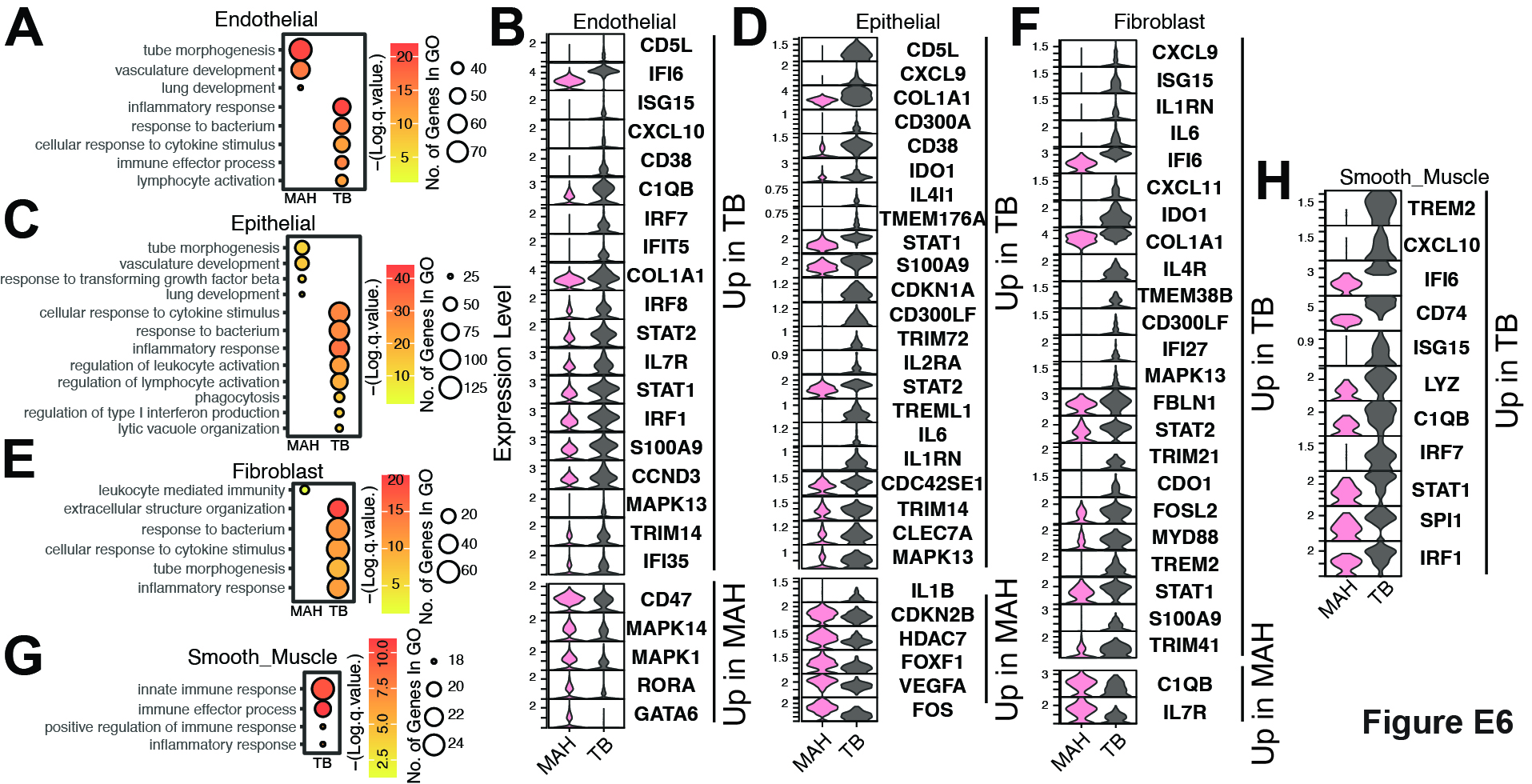

Supplement: Supplementary file 14 [file Image_6.JPEG]
